# Revisiting the relationship between illusory hand ownership induced by visuotactile synchrony and cardiac interoceptive accuracy

**DOI:** 10.1038/s41598-023-43990-2

**Published:** 2023-10-10

**Authors:** Toyoki Yamagata, Kaito Ichikawa, Shogo Mizutori, Yusuke Haruki, Kenji Ogawa

**Affiliations:** 1 Department of Psychology, Hokkaido University, Kita 10, Nishi 7, Kita-Ku, Sapporo, 060-0810 Japan; 2 Department of Behavioral Science, Hokkaido University, Hokkaido University, Kita 10, Nishi 7, Kita-Ku, Sapporo, 060-0810 Japan

**Keywords:** Psychology, Human behaviour

## Abstract

Multisensory integration plays an important role in the experience of the bodily self. Recently, the relationship between exteroception and interoception has been actively debated. The first evidence was a report that the susceptibility of the sense of ownership over a fake hand (i.e., illusory hand ownership: IHO) in the typical rubber hand illusion is negatively modulated by the accuracy of the heartbeat perception (i.e., cardiac interoceptive accuracy: CIA). If reliable, this would suggest an antagonism between the exteroceptive and interoceptive cues underlying the bodily self. However, some inconsistent data have been reported, raising questions about the robustness of the initial evidence. To investigate this robustness, we estimated the extent of the modulatory effect of CIA on IHO susceptibility by applying Bayesian hierarchical modeling to two independent datasets. Overall, our results did not support that IHO susceptibility is modulated by CIA. The present estimates with high uncertainty cannot exclude the hypothesis that the relationship between IHO susceptibility and CIA is so weak as to be negligible. Further studies with larger sample sizes are needed to reach a conclusion about the extent of the modulatory effect. These findings highlight the lack of robustness of key evidence supporting the “antagonism hypothesis”.

## Introduction

The integration of multisensory information in our brain plays an important role in the coherent experience of the self^1–5^. Since Gallagher^6^, the sense of ownership has been considered a fundamental component of the minimal or embodied aspect of the self; at least, the sense of body ownership is thought to be based on a multisensory integration process^7^. Over the past 25 years, that idea regarding body ownership has been demonstrated specifically for a hand (e.g.,^8–15^), using the rubber hand illusion (RHI^16^). In the typical RHI paradigm, a “fake hand,” such as a hand model, is presented to participants while the participant's real hand is hidden (for a review of methodological variations, see^17^). Tactile stimuli are then applied simultaneously and in phase (i.e., synchronously) to the same location on these hands (e.g., the index finger). After this induction, a substantial proportion of participants experienced the fake hand as if it were their own, i.e. illusory hand ownership (IHO). IHO suggests that body ownership can be easily altered through the integration process of exteroceptive information such as vision and touch. Thus, the bodily self based on exteroceptive cues is thought to be highly malleable^1,18,19^.

Interoception, which refers to sensations coming from within the body, such as the cardiovascular system, is also the foundation of the self^1,20–22^. In the empirical science of interoception, the accuracy of heartbeat perception, called cardiac interoceptive accuracy^23^ (CIA), has been frequently investigated by using the heartbeat tracking task^24,25^. Unifying the accumulated findings of CIA within the framework of predictive processing, the following explanation has been proposed^26^: the stability of the predictive model in the brain to control its own physiological states to stay within a narrow and appropriate range for survival underlies the stability of the self. In other words, the bodily self based on interoceptive cues, in contrast with the exteroceptive bodily self, is thought to be highly stable^1,18^.

Tsakiris and colleagues^27^ provided the first empirical finding implying the association between exteroceptive and interoceptive cues in the bodily self. This study reported that individuals with high CIA had a smaller increase in subjective IHO intensity due to classical RHI induction (i.e., synchronous visuotactile stimulation) than individuals with low CIA, suggesting that IHO susceptibility is negatively modulated by CIA. Tsakiris^1^ explains this finding that individuals with accurate heartbeat perception are able to resist the classic rubber hand illusion, including the alteration of hand ownership, because they are anchored in their own bodies by interoceptive signals, suggesting antagonism between exteroceptive and interoceptive cues. However, several inconsistent findings have been reported in recent years^28–31^. For instance, Horváth et al.^29^ showed that there is no modulatory effect of CIA on the susceptibility to the embodiment of a fake hand, which is the RHI experience including IHO. In addition, the correlation analysis failed to support the existence of an association between IHO intensity and CIA. Thus, it is possible that the finding of Tsakiris et al.^27^ is not robust, although it is considered a key piece of empirical evidence in explaining the relationship between the exteroceptive and interoceptive aspects of the bodily self.

Therefore, we aimed to investigate the robustness of the finding that IHO susceptibility is negatively modulated by CIA. To this end, we estimated the extent of the modulatory effect by applying statistical modeling to our unpublished experimental data including the typical RHI paradigm for inducing IHO and the heartbeat tracking task for assessing CIA. Statistical modeling provides more precise estimates and gives us a more plausible view of the relationship between IHO susceptibility and CIA than previous studies. Our experiment is based on Horváth et al.^29^ with modifications in the measurement of IHO and CIA (for details, see “Methods”). We also analyzed a subset of the open data in Horváth et al.^29^ applying the same model to confirm the validity of the results obtained from our data.

We employed a regression model predicting IHO intensity. When regressing IHO on the experimental condition, which is dichotomously coded so that 0 and 1 represent the asynchronous and synchronous stimulation respectively, IHO susceptibility is represented as the value of the slope coefficient. IHO susceptibility is assumed to vary across individuals, and the present study attempts to evaluate the extent to which this variation is explained by CIA. Thus, our model should allow the slope coefficient of the condition to vary across individuals and be predicted by their CIA. We constructed a linear mixed model that satisfies these requirements, which is reduced to the following regression equation:

$$\text{IHO}_{i}^{*} = \left( {\beta_{1} + r_{i} } \right)\text{cond} + \beta_{2} \text{CIA}_{i} + \beta_{3} \text{cond} \times \text{CIA}_{i} + e,$$

where subscription $i$ represents a participant. The outcome variable is the response to a Likert-type questionnaire item with the statement "It seemed like the rubber hand was my hand," and thus the regression needs to be an ordinal one. We chose an ordered-probit regression, which allows us to interpret the left-hand side of the regression equation, ${\text{IHO}}^{*}$, as a normally distributed latent variable behind the manifest ordered response^32,33^. ${r}_{i}$ is the random slope of the participant $i$. This random term $r$ follows a normal distribution with mean zero, namely, ${\text{Normal}}\left(0,{{\sigma }_{r}}^{2}\right)$. The coefficient of the interaction term ${\upbeta }_{3}$ can be interpreted as the slope coefficient in an implicit linear regression of IHO susceptibility on CIA (for details, see “Methods”). If participants with high CIA are less susceptible to RHI induction, this coefficient will be negative. Accordingly, the random term $r$ can be interpreted as the error term in this implicit regression since it represents the unexplained individual difference in IHO susceptibility.

We use Bayesian estimation for model fitting. This choice is motivated by both practical and epistemological reasons. Bayesian statistical modeling is highly flexible, allowing a wide range of models to be tractable by non-statisticians. Furthermore, the evaluation of uncertainty in parameter estimation is made more easily and intuitively in this approach. In contrast to the confidence interval in classical estimation, the posterior distribution, which is the solution of Bayesian estimation, is interpreted as representing the uncertainty in the true value of the parameter without introducing hypothetical sampling or statistical tests. Both the likelihood and the prior distribution affect the posterior distribution. In principle, however, the choice of prior is not unique. While we used weakly informative priors in the analyses reported here, we tried some other choices and found that they made no qualitative difference in the implication (for details, see Supplementary Methods and Results).

## Results

Here, we report only the results of Bayesian statistical modeling of the response data for item 3, "It seemed like the rubber hand was my hand." Distributions of the variables and descriptive statistics of IHO and CIA and estimated results of other items are presented in Supplementary Methods and Results.

### Our data

**Figure 1:**
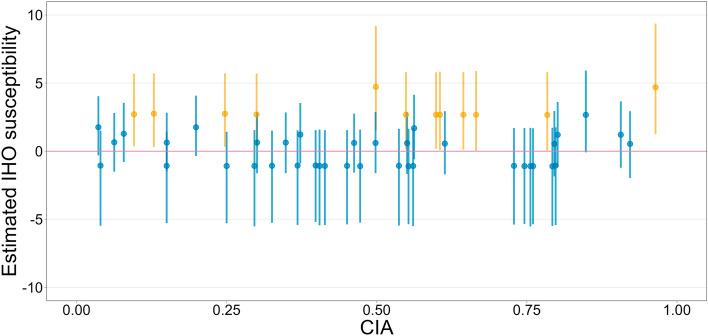
The relationship between the estimated IHO susceptibility (${\upbeta }_{1}+{\upbeta }_{3}{\text{CIA}}_{i}+{r}_{i}$; for why the estimate is represented by this equation, see “Methods”) and CIA in the data collected in our experiment. Each point and vertical line represents the posterior median and the 95% credible interval, respectively, and the color indicates whether the 95% credible interval includes 0 or not (blue, it does).

After confirming convergence, at least for the focal parameters (for details on how the convergence of Markov Chain Monte Carlo was checked, see Supplementary Methods and Results), we evaluated their posterior distributions (Table 1). They indicated that the modulatory effects of CIA on the IHO intensity at baseline and IHO susceptibility for each individual were quite uncertain, ${\upbeta }_{2}$ = 0.26 (95% CrI = [− 0.89, 1.41]) and ${\upbeta }_{3}$ = 0.33 (95% CrI = [− 2.25, 2.98]) (for what each parameter represents, see also the derivation of the regression equation in “Methods”). Figure 1 illustrates the relationship between the estimated IHO susceptibility and CIA. The points are estimates (posterior median) of IHO susceptibility and the vertical segments are their uncertainty (95% CrI). Thus, if CIA has a negative effect on IHO susceptibility, the points and segments should line up in a downward trend (see Fig. 1(c) in Tsakiris et al.^27^). However, Fig. 1 does not show such a trend. These results do not support the idea that individuals with higher CIA are more able to resist the IHO experience.

**Table 1:** Summary of the parameters for our model fitted to the data collected in our experiment.

Parameter	Estimate (posterior median)	95% credible interval
${\upbeta }_{1}$	0.51	[− 0.35, 1.33]
${\upsigma }_{r}$	2.19	[1.12, 3.69]
${\upbeta }_{2}$	0.26	[− 0.89, 1.41]
${\upbeta }_{3}$	0.33	[− 2.25, 2.98]

To further investigate the modulatory effect of CIA on IHO susceptibility, we calculated two effect sizes. One is ${\upbeta }_{3}$ scaled by ${\upsigma }_{r}$ (i.e., the square root of the variance of ${r}_{i}$), which is inspired by the standardized mean difference well-known as “Cohen’s d”. Here, we referred to this as standardized ${\upbeta }_{3}$. Standardized ${\upbeta }_{3}$ represents the standardized difference between the average IHO susceptibilities in a population of individuals with CIA of 0 (i.e., unable to perceive their own heartbeat at all) and that in a population of individuals with CIA of 1 (i.e., able to perceive their own heartbeat perfectly). This statistic, calculated from the estimated ${\upbeta }_{3}$ and ${\upsigma }_{r}$, was also quite uncertain, standardized ${\upbeta }_{3}$ = 0.16 (95% CrI = [− 1.09, 1.40]). This suggests that even when comparing IHO susceptibility between populations with the lowest and highest CIA, we may be observing only a small difference. More importantly, the high uncertainty tells us that the sample size is insufficient and that we should not conclude on the extent of the modulatory effect.

Another effect size is the Bayesian ${R}^{2}$ calculated based on the definition of Gelman and colleagues^34^. It represents the extent to which the variance (i.e., inter-individual variability) in CIA explains that in IHO susceptibility. Although a higher ${R}^{2}$ means that more variance in IHO susceptibility was explained (0 ≤ ${R}^{2}$  ≤ 1), the calculated result was quite small, ${R}^{2}$ = 0.01 (95% HPDI = [1.94 $\times$ 10^−11^, 0.10]). In other words, the fact that IHO susceptibility varies from person to person can hardly be explained by the trait of heartbeat perception.

Note that the estimated fixed effect of condition (${\upbeta }_{1}$), which is the “average” effect at the population level, was also uncertain, ${\upbeta }_{1}$ = 0.51 (95% CrI = [− 0.35, 1.33]). In other words, it is possible that our RHI induction was less effective. This can also be seen in Fig. 1. Inspection of the individual vertical segments reveals that many of them are colored blue, indicating that many participants (38 out of 50) included 0 in the 95% CrI for the estimated IHO susceptibility (although this proportion varies depending on the setting of the prior of ${\upsigma }_{r}$, this variation does not affect the main claim of the present study; for details, see Supplementary Methods and Results).

### Open data

**Figure 2:**
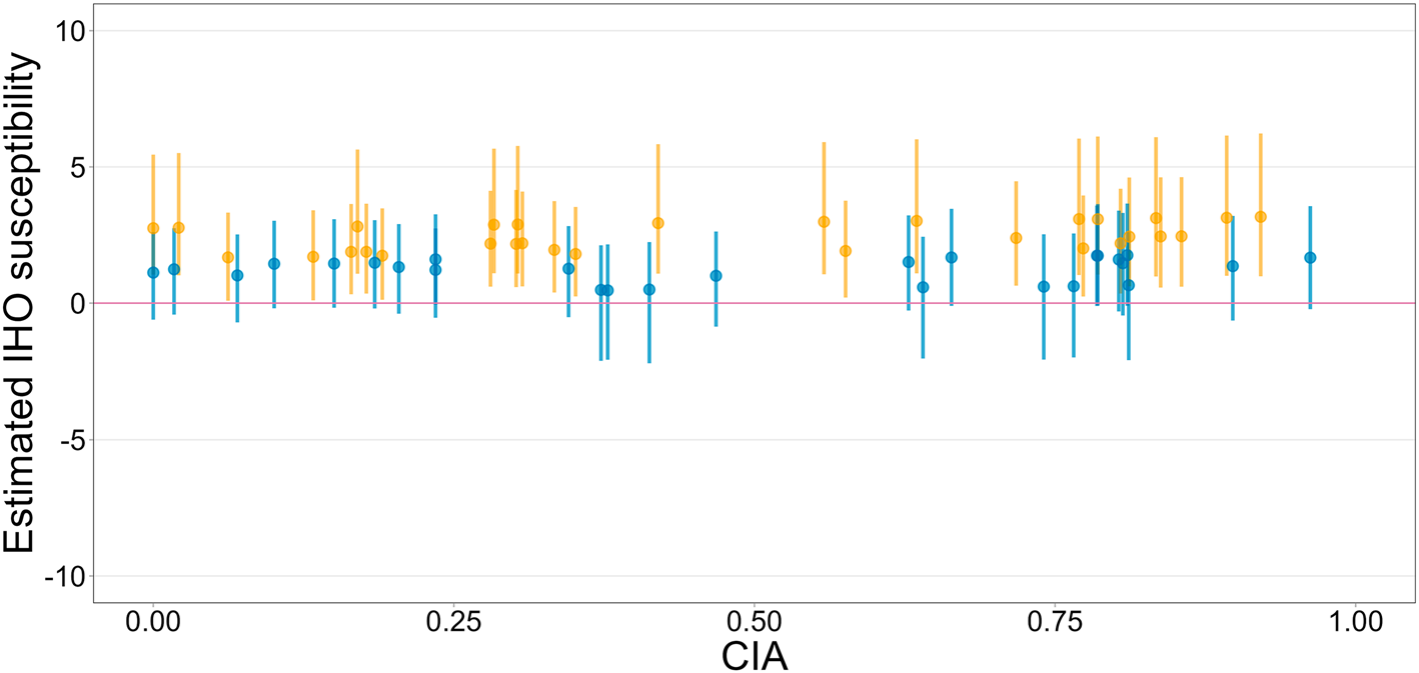
The relationship between the estimated IHO susceptibility (${\upbeta }_{1}+{\upbeta }_{3}{\text{CIA}}_{i}+{r}_{i}$; for why the estimate is represented by this equation, see “Methods”) and CIA in the data collected by Horváth et al.^29^. Each point and vertical line represents the posterior median and the 95% credible interval, respectively, and the color indicates whether the 95% credible interval includes 0 or not (blue, it does).

Again, after confirming convergence, at least for the focal parameters (for details, see Supplementary Methods and Results), we evaluated their posterior distributions (Table 2). First, it is worth noting the estimate of the fixed effect of the condition, ${\upbeta }_{1}$ = 1.75 (95% CrI = [1.20, 2.43]). Horváth et al.^29^ seem to have succeeded in inducing stronger IHO experiences in their participants on average. This can also be seen in Fig. 2, which was generated in the same way as Fig. 1. Inspection of the individual vertical lines reveals that many of them are colored orange, indicating that more than half of the participants (31 out of 58) did not include 0 in the 95% CrI for estimated IHO susceptibility. In addition, we should also focus on the estimated square root of the variance of ${r}_{i}$, ${\upsigma }_{r}$ = 1.17 (95% CrI = [0.28, 2.10]). Compared to the estimate in our data, ${\upsigma }_{r}$ = 2.19 (95% CrI = [1.12, 3.69]), the inter-individual variability in the RHI induction effect was smaller in the data of Horváth et al.^29^. All this implies that the RHI induction of Horváth et al.^29^ may be more effective than ours as a manipulation to induce IHO.

**Table 2:** Summary of the parameters for our model fitted to the data collected by Horváth et al.^29^.

Parameter	Estimate (posterior median)	95% credible interval
${\upbeta }_{1}$	1.75	[1.20, 2.43]
${\upsigma }_{r}$	1.17	[0.28, 2.10]
${\upbeta }_{2}$	− 0.60	[− 1.49, 0.29]
${\upbeta }_{3}$	0.28	[− 1.29, 1.85]

Nevertheless, the modulatory effects of CIA on the baseline IHO intensity and IHO susceptibility were found to be quite uncertain, ${\upbeta }_{2}$ =  − 0.60 (95% CrI = [− 1.49, 0.29]) and ${\upbeta }_{3}$ = 0.28 (95% CrI = [− 1.29, 1.85]). Consistent with this, Fig. 2 shows no trend, similar to the results of our data. Furthermore, according to standardized ${\upbeta }_{3}$, even the sample size of the previous study also is not sufficient to conclude the extent of the modulatory effect, standardized ${\upbeta }_{3}$ = 0.25 (95% CrI = [− 1.24, 2.37]). According to another effect size ${R}^{2}$, IHO susceptibility could not be explained by CIA, ${R}^{2}$ = 0.02 (95% HPDI = [2.69 $\times$ 10^−12^, 0.25]). In summary, the analysis of the open data yielded the same findings as the analysis of our data, except for the average effect of RHI induction at the population level.

## Discussion

The aim of our study is to investigate the robustness of the finding that IHO susceptibility is negatively modulated by CIA. To this end, we analyzed unpublished data from our experiment including a typical RHI experiment and a heartbeat tracking task based on Horváth et al.^29^, and estimated the extent of this modulatory effect using Bayesian statistical modeling. In addition, to confirm the validity of the results obtained from our original data, we analyzed a subset of the data in Horváth et al.^29^ using the same procedure. Overall, our results did not support that IHO susceptibility is modulated by CIA.

This negative finding of our study is not surprising. We conducted a search of previous articles citing Tsakiris et al.^27^ and containing the term “rubber hand illusion” in Web of Science (https://www.webofknowledge.com) on 24th August 2022. Although this search returned 93 articles (Supplementary Data), there were no reports that the IHO result of Tsakiris et al.^27^ had been rigorously replicated. It has been more than 10 years since the issue of reproducibility or replicability began to receive attention in scientific research. This is an extremely serious problem, especially in psychology as a scientific discipline^35^. In RHI research, direct/conceptual replication studies have begun to be conducted in recent years (e.g.,^30,36,37^), and sometimes, results that differ negatively from previous studies have been reported^30^. We should actively confirm the reproducibility of key findings in each research topic. To better understand the relationship between IHO susceptibility and CIA, direct replication of Tsakiris et al.^27^ and meta-analysis are required in the future.

The results of the present study highlight the need to revisit the relationship between the exteroceptive and interoceptive cues underlying the bodily self. As shown above, there is no empirical evidence to support the view proposed by Tsakiris^1^ that the two cues in the bodily self are antagonistic. However, Tsakiris et al.^27^ is not the only study that offers suggestions about the relationship between body ownership and interoception. In particular, we should pay attention to the well-known study by Suzuki and colleagues. Suzuki et al.^38^ created a special RHI paradigm, a method of presenting a virtual hand that flashes in synchrony or asynchrony with the participant’s heartbeat. As a result, a virtual hand illusion was induced in the synchronous condition. Subsequent studies extending such “cardiovisual stimuli” for the full-body illusion have yielded consistent results^39,40^, that is, on average, participants experienced illusory body ownership when the body flashed in synchrony with their own heartbeats. A review of these findings suggests that there is a single neural system that processes both exteroceptive and interoceptive signals, and that these bodily signals (especially torso-related information) jointly form the basis of bodily self-consciousness^41^. Thus, the relationship between exteroception and interoception in the bodily self may not be antagonistic, but rather flexible and complementary.

Then, why did Tsakiris et al.^27^ observe the negative association between IHO susceptibility and CIA? Perhaps multiple factors are at play, but one possibility is that there is a third (latent) factor that influences both IHO and CIA, leading to a pseudo-correlation. For example, top-down factors such as task expectations conveyed by demand characteristics or based on knowledge have been of concern and discussed for their influence on IHO and CIA, respectively^23,30,42,43^. If these top-down factors could positively modulate IHO and negatively modulate CIA, a spurious negative association between IHO and CIA might be confirmed in some populations. However, recent IHO studies suggest that the influence of the task expectation is not dominant, but rather depends more strongly on the multisensory integration process in the brain^44^. Heart rate knowledge is also thought to have rather an upward effect on CIA^45^. Nevertheless, the possibility of other confounding factors cannot be excluded. Future studies may be needed to investigate such candidate confounders in order to identify and control them.

How to measure IHO and CIA also needs to be discussed. In the present study, we used the most commonly used indices for both IHO and CIA, that is, the rating on a Likert-type questionnaire item and the score on the heartbeat tracking task. However, the validity of these indicators has been questioned in recent years (e.g., IHO: Chancel and Ehrsson^46^; CIA: Ferentzi et al.^23^). The widespread acceptance (with misunderstanding) of the finding of Tsakiris et al.^27^ and the claim of Tsakiris^1^ may be due in part to the interpretation that includes results from different measures (i.e., proprioceptive drift) that may not directly measure IHO. For instance, a recent systematic review^47^ with meta-analysis also argues that proprioceptive drift is weakly related to IHO or the subjective embodiment of a fake hand. Therefore, in the future, it will be necessary to discuss the relationship between IHO susceptibility and CIA after appropriately categorizing the findings by the difference in measurements, as shown in the present study. In addition, more accurate methods of measuring IHO and CIA need to be explored. In their respective fields of research, active attempts to develop more accurate measurements have already begun (e.g., IHO: Lanfranco et al.^48^; CIA: Pohl et al.^49^). However, there is no established method yet, and continued research for better measurements is required.

A limitation of the present study is that the RHI induction appeared to be less effective in our experiment. As shown in Fig. 1, the proportion of participants who unambiguously experienced IHO in response to our RHI induction (12 out of 50, or 24%) was considerably low. This problem is pronounced when compared to the corresponding results of the secondary analysis (31 out of 58, or 53%) as shown in Fig. 2. Although it is impossible to determine the reason for this discrepancy, it may be due to the material of the fake hand. The fake hand used in our experiment was a silicone product without a skeleton. As a result, the fingers were so soft that we cannot rule out the possibility that they moved unnaturally during the brush strokes. Also, the use of the gloves in order to control the feature as a visual stimulus by eliminating the differences in appearance between the two “hands” may have contributed to the questionable effectiveness of our induction method. We used thick string knit gloves because they were easy to put on the fake hand. This choice would have weakened the intensity of the tactile stimuli and interfered with the synchrony of the stimulation. In addition, the gloved fake hand would not have been as natural as using a more sophisticated prosthetic hand. In light of the above, there is an urgent need to standardize the methodology for RHI experiments, such as materials, the setup, and the stimulation procedure. First, the methods should be reported in as much detail as possible in each article. Furthermore, automation of the illusion induction^50^ is one of the prospective future directions.

Another limitation and pressing issue is the insufficient sample size. Our results showed that the two datasets we analyzed were insufficient to estimate the extent of the modulatory effect of CIA on IHO susceptibility. The sample size of Tsakiris et al.^27^ (*N* = 46) was even smaller than these datasets. A “significant difference” found in a study with an insufficient sample size is less likely to reflect a true effect and is more likely to be an extreme estimate that deviates from the true difference^51^. The latter problem, in particular, is known as the “winner’s curse”. In addition, the tendency for the first published study to often be the most cursed is called the Proteus phenomenon. The results of the present study suggest that Tsakiris et al.^27^ may be a case of this phenomenon. The current widespread acceptance of the finding of Tsakiris et al.^27^ may tell us that, unfortunately, we are often fascinated by cursed winners. In the future, the goal of inferential statistics must first be clarified, and then an appropriate design analysis should be performed for that purpose.

The present statistical model of quantifying inter-individual variability in IHO susceptibility as a parameter of random effects in an ordinal regression model is a novel approach not seen in previous studies. To our knowledge, the previous studies have quantified IHO susceptibility by the arithmetic operation of taking the difference between synchronous and asynchronous conditions of raw ratings (e.g.^27^) on a single Likert-type question item or a scale score (e.g.^29^), which is the average of ratings on multiple question items. However, it has been pointed out that the results can be distorted (and in some cases reversed) by analyzing ordinal data such as a Likert-type response as continuous^32^. In addition to this fundamental problem, the methods of previous studies provide IHO susceptibility as a constant and fail to take its uncertainty into account in the estimation. If uncertainty is not properly incorporated, parameters of interest may be overestimated or underestimated^52^. In contrast, the present study provided new possibilities for more accurate quantification of IHO susceptibility. In this respect, the present study may provide more plausible evidence that the inter-individual variability in IHO susceptibility can hardly be explained by the individual difference in CIA.

In summary, our investigation has shed light on the lack of robustness of key evidence supporting the hypothesis of antagonism between the exteroceptive and interoceptive cues underlying the bodily self by applying a novel analytic approach to two independent datasets. None of the results of the present study were consistent with the finding of the previous study. Rather, the estimated effects of CIA on IHO susceptibility in our study had large uncertainties, and thus our findings cannot rule out the hypothesis that the relationship between IHO susceptibility and CIA is so weak as to be negligible. We should not yet draw a conclusion based on the data with insufficient sample sizes. It may be time for us to revisit the accumulated previous findings for a scientific understanding of the mechanism of the bodily self.

## Methods

The methods of our study are described below, along with a partial description of the methods of Horváth et al.^29^ that are relevant to our study. Details of the latter can be found in the original paper.

### Participants

Sixty healthy volunteers (30 males and 30 females) participated in our experiment. The number of participants was determined based on Horváth et al.^29^. In this previous study, the number of participants (*N* = 60, 53% female, 87% right-handed) was determined based on a priori sample size calculation. The mean age of the 59 participants was 21.14 years (*SD* = 1.12, 19–24 years), excluding one participant who gave an obviously incorrect age response. All participants had normal or corrected-to-normal visual acuity and self-reported right-handedness. Note that some of them, when asked using a modified version of the Edinburgh Handedness Inventory^53^, reported that they had received a dominant hand correction in the past, and/or that they sometimes or frequently used their left hand in certain situations (e.g., when using a screwdriver or brushing their teeth).

Ten participants were those who knew about the RHI prior to participating in the experiment (RHI-known participants) and/or those who had participated in other RHI experiments (RHI-experienced participants). It is possible that for these participants, the expectation that the RHI would occur may have a powerful influence on the experiment results. Therefore, we have excluded these data from the subsequent analysis.

We used data from 50 naïve participants in the subsequent analysis. Horváth et al.^29^ do not appear to have excluded data according to the criteria used in our study (naïve or not). Instead, other technical issues (missing value in questionnaire data) led to the exclusion of data from 2 out of 60 participants. Thus, the secondary analysis in our study included data from 58 individuals who participated in Horváth et al.^29^.

### Procedure

Our experimental protocol was approved by the Ethical Committee of the Center for Experimental Research in Social Sciences (CERSS) of the Hokkaido University. The entire experiment lasted approximately 60 min.

Prior to the experiment, participants received a brief verbal explanation of the background, purpose, and methods of the study and completed a written informed consent for participation in the experiment, which was conducted in accordance with the guidelines of CERSS. The explanation was limited to the following: “What you are going to do now is a brush tracing observation task. I will stroke the fake hand and your left hand with the brush, and you are to observe the fake hand being stroked with the brush.” Thus, it is assumed that participants other than the RHI-known and RHI-experienced participants were naïve and did not understand the true purpose of this task or the intention of the experimental manipulation. However, they were fully informed in advance that the experiment would not involve any risks, and their consent was obtained.

After giving informed consent, participants were asked to complete a web-based questionnaire that asked for basic information (e.g., name and date of birth) and a questionnaire that asked about their previous experience playing musical instruments (especially, the piano) and exercising. However, this information is beyond the scope of this study and will not be discussed here.

After completing the questionnaires, three types of tasks (fingertip position estimation, heartbeat tracking, and RHI) were administered to each participant in a randomized order. Here, we focus on the RHI and heartbeat tracking tasks and omit the explanation of the fingertip position estimation task.

#### RHI task

**Figure 3:**
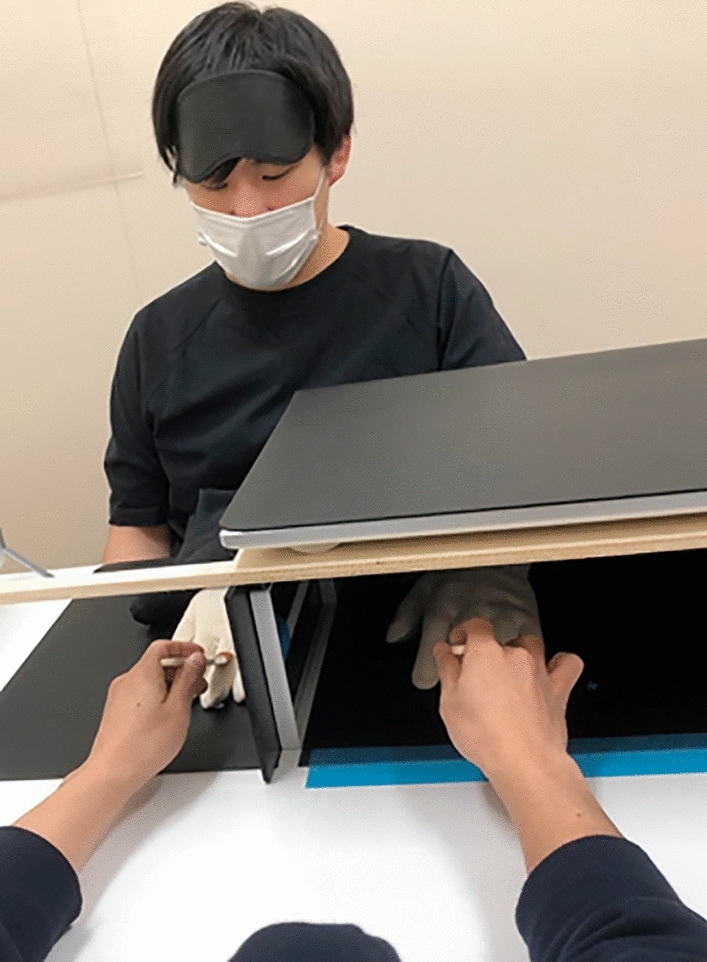
The setup of the RHI experiment.

Our RHI task had a within-participant one-factorial design (visuotactile stimulation: synchronous vs. asynchronous). Participants performed the task in the setup shown in Fig. 3.

- Setup

First, participants aligned their body midline to a predetermined position according to the experimenter’s instructions. After the participants sat down, the experimenter instructed the participants about the task. Then, the participant put on a glove on the left hand and an eye mask, and placed the left arm in a box placed on the desk. The box was designed so that the participant’s left hand was not visible, and was used to hide the “real” left hand. A touch display (LL-S242A-W, SHARP) was attached to the top of the box and was used to measure and record the participants’ responses. Next, the experimenter moved the real hand so that the fingertip of the middle finger met a predetermined position (20 cm to the left of the participant’s midline). Participants then estimated the position of the (hidden) middle fingertip of their left hand while wearing an eye mask and with their eyes closed. This procedure was used to measure the baseline of the proprioceptive drift.

The experimenter then placed a silicone left-hand model in front of the participant, with the middle finger aligned with the participant’s midline. The position of this fake hand was adjusted to be parallel to the participant’s actual left hand. The fake hand was pre-fitted with a glove identical to the one worn by the participant. This was done to control for the nature of the visual stimuli by eliminating differences in appearance (e.g., surface texture and coloration) between the real hand and the fake hand. In addition, since the fake hand is constructed with almost no arm part, there was concern that the appearance of the cross-section would cause discomfort. Therefore, we covered the wrists of the fake hand with a black towel.

Next, participants were instructed to remove the eye mask with their right hand only, without moving their left hand. However, they were asked to keep the mask on their forehead (Fig. 3), not to remove it completely, to facilitate the multiple applications and removals of the mask in subsequent procedures. With the fake hand clearly visible to the participant, the experimenter presented synchronous or asynchronous stimuli in the manner described in detail below.

- Conditions

In the synchronous condition, the experimenter stroked only the middle fingers of both the fake hand and the real hand at approximately the same location and time. The strokes lasted for 90 seconds per block (i.e., per condition). This stimulus duration was the same as in the experiment of Horváth et al.^29^. The experimenter listened to the beeps from the PC through an earphone while performing the brush strokes according to the timing of the beeps. The beeps were generated using MATLAB R2019a (MathWorks, Natick, USA). Three pseudo-random jitters (−200 ms, 0 ms, and +200 ms) were included to average 1 second per interval between beeps. This was done to make the stimulus series as unpredictable as possible for the participants. Riemer et al.^17^ pointed out that no studies have yet directly demonstrated the effect of such a treatment on the RHI, but argued that it has some influence on the results. Because there seemed to be a belief that such treatments could induce a stronger RHI (e.g.^54,55^), we introduced jitter into the stimulus series. The sequence of this jitter was programmed to be different for each participant. Immediately after 90 s of brushing, participants responded to the guessed position of the middle fingertip of the left hand using a procedure similar to that described above as a baseline measure of proprioceptive drift. At this time, participants wore an eye mask with their right hand only, without moving their left hand. After all the proprioceptive drift measures were completed, the participants continued to answer the question items measuring the intensity of subjective experiences of RHI using the touch display on the box. PsychoPy^56^ (v3.2.4) was used to present the question items and to record the responses.

In also the asynchronous condition (i.e., the control condition), the brush strokes were applied only to the real hand and the middle finger of the fake hand. However, in the asynchronous condition, the phase of the strokes on each “hand” was shifted by 180°. In other words, when the brush stroke on the real hand was on the fist side, the brush stroke on the fake hand was on the fingertip side. Other than this difference in stroking (i.e., the duration of a block, the method of determining the timing of the stroke, and the pre- and post-stimulus procedures), everything was the same as in the synchronous condition.

The order of the two conditions was randomized across participants. During the stimulus presentation and the measurement of proprioceptive drift, participants were instructed to place their left hand and arm on the desk in a completely relaxed position and were not allowed to move them. However, they were allowed to move their left hand during inter-block (i.e., between conditions) breaks and during subjective rating. They were also allowed to temporarily remove their gloves during these breaks.

- Measurement

The questionnaire for the subjective ratings after each condition consisted of seven items used in Horváth et al.^29^ and translated into Japanese by us (Table 3). All items were Likert-type questions with a 7-point scale from −3 to +3 (−3 = “not at all agree”, 0 = “neither agree nor disagree”, +3 = “strongly agree”). Note that although in Horváth et al.^29^ all items had an 11-point scale (1 = “strongly disagree” to 11 = “strongly agree”), we modified it to be a more general Likert item as the RHI measure. The order in which the items were presented was randomized for each participant. These items were drawn from the 27 items used in Longo et al.^57^.

**Table 3:** Summary of the items used in our study and the constructs they measure.

Item #	Statement (Our experiment)	What to measure (Horváth et al., 2020)	What to measure (Longo et al., 2008)	What to measure (Romano et al., 2021)
1	It seemed like I was feeling the touch of the paintbrush in the location where I saw the fake hand being touched	Embodiment (Referral of touch)	?	?
2	It seemed like the touch I felt was caused by the paintbrush touching the fake hand	Embodiment (Referral of touch)	Embodiment of rubber hand (Location)	Embodiment
3	It seemed like the fake hand was my hand	Embodiment (Ownership)	Embodiment of rubber hand (Ownership)	Embodiment
4	It seemed like the fake hand belonged to me	Embodiment (Ownership)	Embodiment of rubber hand (Ownership)	Embodiment
5	It seemed like I was unable to move my hand	Disembodiment (Loss of agency)	Loss of own hand	Disembodiment
6	It seemed like my hand had disappeared	Disembodiment (Loss of hand position)	Loss of own hand	Disembodiment
7	It seemed like my hand was out of control	Disembodiment (Loss of agency)	Loss of own hand	Disembodiment

7-point Likert-type items (− 3 = “not at all agree” to + 3 = “strongly agree”, and 0 = “neither agree nor disagree”).

Horváth et al.^29^ further divided these seven items into the following two categories in terms of the concepts they measure: four items measuring the embodiment of the fake hand and three items measuring the disembodiment of the real hand (see also Table 3). In the present study, however, no such categorization was made, and only the data from item 3 (“it seemed like the fake hand was my hand”) were analyzed. The reason for this is that the validity of the two categories in Horváth et al.^29^ was questionable. The question of the structure of the subjective RHI experience is still under debate, and although there are some research findings (e.g.,^57,58^), there is no established theory. In the first place, the method of using the arithmetic mean of multiple items as a “scale” assumes (even implicitly) that some constructive concepts behind the observations can be measured by adding the ratings of these items with the same “weight”. However, it is not clear that such an assumption is valid. Therefore no arithmetic averaging was performed in the present IHO measurement.

Item 3 is the most widely used to measure IHO. The above proposal by Tsakiris^1^ may be based on the result of the analysis of rating data for this statement in Tsakiris et al.^27^. Almost all of the other question items and more “implicit” indicators (i.e., proprioceptive drift) commonly used to measure RHI intensity do not measure IHO, at least not directly^57,59–61^. Therefore, our discussion is essentially based on the results of analyzing the data for this item only.

#### Heartbeat tracking task

In the heartbeat tracking task, the accuracy of the heartbeat perception is assessed by evaluating the discrepancy between the perceived heartbeat and the measured heartbeat. The task in our study is based on the procedure used by Haruki & Ogawa^62^.

After participants were seated in a chair, a pulse oximeter (IWS920, Tokyo Devices) was placed on the left index finger. The experimenter verbally instructed the participants as follows: “You will hear two whistles from this computer, so start counting after the first whistle and stop counting after the second whistle. After the second whistle, I will verbally ask you how many times you have counted. When counting, do not take the pulse with your hand, but focus on the beat and sound from your heart. Count only the number of times you perceive some kind of change, such as a feeling of movement around the heart or the sound of a heartbeat. Even if the number is zero, it does not matter. Please answer according to your perception.” The second half of this instruction was designed to ensure that participants did not estimate the number of heartbeats, as this would influence CIA^45,63^. The task trials were performed at least 1 min after the pulse oximeter was attached, to control the level of arousal. Participants performed three trials of different duration (25, 35, and 45 s; 25, 35, and 55 s in Horváth et al.^29^) after a short training trial (10 s). The order of the trials was randomized for each participant, with a rest period of at least 30 s between each trial.

Individual CIA was calculated using the following formula^27,29,64^:

$${\text{CIA}}_{i} = \frac{1}{3}\sum\nolimits_{t = 1}^{3} {\left( {1 - \frac{{\left| {{\text{HB}}_{{{\text{rec}},t}} - {\text{HB}}_{{{\text{rep}},t}} } \right|}}{{{\text{HB}}_{{{\text{rec}},t}} }}} \right)} ,$$

where ${\text{CIA}}_{i}$ represents CIA of participant $i$. HB_rec_ is the number of heartbeats estimated from the recorded pulses, while HB_rep_ is the number of heartbeats reported by the participants. And, the subscript $t$ represents the trial number. Thus, CIA ranges from 0 to 1, with higher values indicating participants can accurately perceive their heartbeat.

### Statistical modeling

We used a regression model to estimate the extent of the modulatory effect of CIA on IHO susceptibility (see “Introduction”). Since our outcome variable is the single-item Likert-type response, which is an ordinal scale^32^, an ordered-probit regression model with the Bayesian method for model fitting (for details, see^33^) was employed. The regression equation is derived in the following way. First, consider a regression of IHO intensity on the experimental condition. This is represented by the following equation:

$${\text{IHO}}^{*}_{i} = {\updelta }_{0,i} + {\updelta }_{1,i} {\text{cond}} + e.$$

Second, since CIA can affect the baseline of IHO intensity, ${\updelta }_{0,i}$ should be a function of CIA:

$${\updelta }_{0,i} = {\upbeta }_{2} {\text{CIA}}_{i} .$$

Third, and more importantly, since we assume that IHO susceptibility, which is represented by ${\updelta }_{1,i}$, varies across individuals, and the present study attempts to evaluate the extent to which this variation is explained by CIA, we consider a regression of the term on CIA:

$${\updelta }_{1,i} = {\upbeta }_{1} + {\upbeta }_{3} {\text{CIA}}_{i} + r_{i} ,$$

where ${r}_{i}$ is a normally distributed error. Assigning the last two equations to the first yields the regression equation given in “Introduction”. Model fittings were implemented using the Markov Chain Monte Carlo algorithm via the brms package^65,66^ in R. For each model fitting, 4 chains were run in parallel (4000 iterations per chain, and warmup = 2000). We used a weakly informative prior (e.g., normal distribution with location = 0 and scale = 2.5 for coefficients). Four distributions were considered as candidates for the prior of ${\sigma }_{r}$. Based on summaries and visualizations of these posteriors (see Supplementary Methods and Results), the half-Cauchy distribution with location = 0 and scale = 2.5 was employed, and only the results are reported here. We confirmed that $\widehat{R}$ of all parameters did not exceed 1.1 to assess the convergence. In addition to the $\widehat{R}$ values, the traceplots of the focal parameters were also checked.

### Supplementary information

Supplementary materials for the preprint of the present study available at https://osf.io/spqwc/.

### Supplementary Information

Supplementary Information 1.

Supplementary Information 2.

## Data Availability

The dataset generated by our experiment will not be made publicly available, as informed consent for that was not obtained. Requests for access to the datasets should be addressed to the corresponding author. The publicly available dataset generated by Horváth et al.^29^ can be found at https://doi.org/10.1016/j.cortex.2020.08.026.

## References

[1] Tsakiris M (2017). The multisensory basis of the self: From body to identity to others. Q. J. Exp. Psychol..

[2] Apps MAJ, Tsakiris M (2014). The free-energy self: A predictive coding account of self-recognition. Neurosci. Biobehav. Rev..

[3] Limanowski J, Blankenburg F (2013). Minimal self-models and the free energy principle. Front. Hum. Neurosci..

[4] Blanke O (2012). Multisensory brain mechanisms of bodily self-consciousness. Nat. Rev. Neurosci..

[5] Blanke O, Slater M, Serino A (2015). Behavioral, neural, and computational principles of bodily self-consciousness. Neuron.

[6] Gallagher S (2000). Philosophical conceptions of the self: Implications for cognitive science. Trends Cogn. Sci..

[7] Ehrsson, H. H. Multisensory processes in body ownership. In *Multisensory Perception: From Laboratory to Clinic* (eds. Sathian, K. & Ramachandran, V. S.) 179–200 (Academic Press; Elsevier, 2020).

[8] Armel KC, Ramachandran VS (2003). Projecting sensations to external objects: Evidence from skin conductance response. Proc. Biol. Sci..

[9] Ehrsson HH, Spence C, Passingham RE (2004). That’s my hand! Activity in premotor cortex reflects feeling of ownership of a limb. Science.

[10] Samad M, Chung AJ, Shams L (2015). Perception of body ownership is driven by Bayesian sensory inference. PLoS One.

[11] Litwin P, Zybura B, Motyka P (2020). Tactile information counteracts the attenuation of rubber hand illusion attributable to increased visuo-proprioceptive divergence. PLoS One.

[12] Chancel M, Hasenack B, Ehrsson HH (2021). Integration of predictions and afferent signals in body ownership. Cognition.

[13] Hsu T-Y, Zhou J-F, Yeh S-L, Northoff G, Lane TJ (2022). Intrinsic neural activity predisposes susceptibility to a body illusion. Cereb. Cortex Commun..

[14] Chancel M, Iriye H, Ehrsson HH (2022). Causal inference of body ownership in the posterior parietal cortex. J. Neurosci..

[15] Chancel M, Ehrsson HH, Ma WJ (2022). Uncertainty-based inference of a common cause for body ownership. Elife.

[16] Botvinick M, Cohen J (1998). Rubber hands ‘feel’ touch that eyes see. Nature.

[17] Riemer M, Trojan J, Beauchamp M, Fuchs X (2019). The rubber hand universe: On the impact of methodological differences in the rubber hand illusion. Neurosci. Biobehav. Rev..

[18] Allen, M. & Tsakiris, M. The body as first prior: Interoceptive predictive processing and the primacy of self-models. In *The Interoceptive Mind: From Homeostasis to Awareness* (eds. Tsakiris, M. & De Preester, H.) 27–45 (Oxford Academic, 2018).

[19] Quigley KS, Kanoski S, Grill WM, Barrett LF, Tsakiris M (2021). Functions of interoception: From energy regulation to experience of the self. Trends Neurosci..

[20] Seth AK, Suzuki K, Critchley HD (2012). An interoceptive predictive coding model of conscious presence. Front. Psychol..

[21] Seth AK (2013). Interoceptive inference, emotion, and the embodied self. Trends Cogn. Sci..

[22] Seth AK, Friston KJ (2016). Active interoceptive inference and the emotional brain. Philos. Trans. R. Soc. Lond. B Biol. Sci..

[23] Ferentzi E, Wilhelm O, Köteles F (2022). What counts when heartbeats are counted. Trends Cogn. Sci..

[24] Schandry R (1981). Heart beat perception and emotional experience. Psychophysiology.

[25] Garfinkel SN, Seth AK, Barrett AB, Suzuki K, Critchley HD (2015). Knowing your own heart: Distinguishing interoceptive accuracy from interoceptive awareness. Biol. Psychol..

[26] Seth AK, Tsakiris M (2018). Being a beast machine: The somatic basis of selfhood. Trends Cogn. Sci..

[27] Tsakiris M, Tajadura-Jiménez A, Costantini M (2011). Just a heartbeat away from one’s body: Interoceptive sensitivity predicts malleability of body-representations. Proc. Biol. Sci..

[28] Crucianelli L, Krahé C, Jenkinson PM, Fotopoulou AK (2018). Interoceptive ingredients of body ownership: Affective touch and cardiac awareness in the rubber hand illusion. Cortex.

[29] Horváth Á (2020). Proprioception but not cardiac interoception is related to the rubber hand illusion. Cortex.

[30] Critchley HD, Botan V, Ward J (2021). Absence of reliable physiological signature of illusory body ownership revealed by fine-grained autonomic measurement during the rubber hand illusion. PLoS One.

[31] Dobrushina OR (2021). Sensory integration in interoception: Interplay between top-down and bottom-up processing. Cortex.

[32] Liddell TM, Kruschke JK (2018). Analyzing ordinal data with metric models: What could possibly go wrong?. J. Exp. Soc. Psychol..

[33] Bürkner P-C, Vuorre M (2019). Ordinal regression models in psychology: A tutorial. Adv. Methods Pract. Psychol. Sci..

[34] Gelman A, Goodrich B, Gabry J, Vehtari A (2019). R-squared for Bayesian regression models. Am. Stat..

[35] Open Science Collaboration (2015). Estimating the reproducibility of psychological science. Science.

[36] Nitta H, Tomita H, Zhang Y, Zhou X, Yamada Y (2018). Disgust and the rubber hand illusion: a registered replication report of Jalal, Krishnakumar, and Ramachandran (2015). Cogn. Res. Principles Implications.

[37] Reader AT (2022). What do participants expect to experience in the rubber hand illusion? A conceptual replication of Lush (2020). Collabra Psychol..

[38] Suzuki K, Garfinkel SN, Critchley HD, Seth AK (2013). Multisensory integration across exteroceptive and interoceptive domains modulates self-experience in the rubber-hand illusion. Neuropsychologia.

[39] Aspell JE (2013). Turning body and self inside out: Visualized heartbeats alter bodily self-consciousness and tactile perception. Psychol. Sci..

[40] Heydrich L (2018). Cardio-visual full body illusion alters bodily self-consciousness and tactile processing in somatosensory cortex. Sci. Rep..

[41] Park HD, Blanke O (2019). Coupling inner and outer body for self-consciousness. Trends Cogn. Sci..

[42] Lush P (2020). Demand characteristics confound the rubber hand illusion. Collabra Psychol..

[43] Lush P (2020). Trait phenomenological control predicts experience of mirror synaesthesia and the rubber hand illusion. Nat. Commun..

[44] Slater M, Ehrsson HH (2022). Multisensory integration dominates hypnotisability and expectations in the rubber hand illusion. Front. Hum. Neurosci..

[45] Desmedt O, Luminet O, Corneille O (2018). The heartbeat counting task largely involves non-interoceptive processes: Evidence from both the original and an adapted counting task. Biol. Psychol..

[46] Chancel M, Ehrsson HH (2020). Which hand is mine? Discriminating body ownership perception in a two-alternative forced-choice task. Atten. Percept. Psychophys..

[47] Tosi G, Mentesana B, Romano D (2023). The correlation between proprioceptive drift and subjective embodiment during the rubber hand illusion: A meta-analytic approach. Q. J. Exp. Psychol..

[48] Lanfranco RC, Chancel M, Ehrsson HH (2023). Quantifying body ownership information processing and perceptual bias in the rubber hand illusion. Cognition.

[49] Pohl A (2021). Cardiac interoception: A novel signal detection approach and relations to somatic symptom distress. Psychol. Assess..

[50] Sivasubramaniam AK, Ng J-H, Chan H, Yang JKY, Kalckert A (2022). The super-stroker—An open-source tool to induce the rubber hand illusion. Psychology of Consciousness: Theory, Research, and Practice.

[51] Button KS (2013). Power failure: Why small sample size undermines the reliability of neuroscience. Nat. Rev. Neurosci..

[52] McElreath R (2020). Statistical Rethinking: A Bayesian Course with Examples in R and Stan.

[53] Oldfield RC (1971). The assessment and analysis of handedness: The Edinburgh inventory. Neuropsychologia.

[54] Limanowski J, Lutti A, Blankenburg F (2014). The extrastriate body area is involved in illusory limb ownership. Neuroimage.

[55] Limanowski J, Blankenburg F (2015). Network activity underlying the illusory self-attribution of a dummy arm. Hum. Brain Mapp..

[56] Peirce J (2019). PsychoPy2: Experiments in behavior made easy. Behav. Res. Methods.

[57] Longo MR, Schüür F, Kammers MPM, Tsakiris M, Haggard P (2008). What is embodiment? A psychometric approach. Cognition.

[58] Romano D, Maravita A, Perugini M (2021). Psychometric properties of the embodiment scale for the rubber hand illusion and its relation with individual differences. Sci. Rep..

[59] Abdulkarim Z, Ehrsson HH (2016). No causal link between changes in hand position sense and feeling of limb ownership in the rubber hand illusion. Atten. Percept. Psychophys..

[60] Reader AT, Trifonova VS, Ehrsson HH (2021). The relationship between referral of touch and the feeling of ownership in the rubber hand illusion. Front. Psychol..

[61] Rohde M, Luca M, Ernst MO (2011). The rubber hand illusion: Feeling of ownership and proprioceptive drift. Do not go hand in hand. PLoS One.

[62] Haruki Y, Ogawa K (2021). Role of anatomical insular subdivisions in interoception: Interoceptive attention and accuracy have dissociable substrates. Eur. J. Neurosci..

[63] Ring C, Brener J (2018). Heartbeat counting is unrelated to heartbeat detection: A comparison of methods to quantify interoception. Psychophysiology.

[64] Pollatos O (2008). Reduced perception of bodily signals in anorexia nervosa. Eat. Behav..

[65] Bürkner P-C (2017). brms: An R package for Bayesian multilevel models using stan. J. Stat. Softw..

[66] Bürkner P-C (2018). Advanced Bayesian multilevel modeling with the R package brms. R J..

