# Supplementary material for: Revisiting the relationship between illusory hand ownership induced by visuotactile synchrony and cardiac interoceptive accuracy
Source: Sci Rep. 2023 Oct 10;13:17132. doi: 10.1038/s41598-023-43990-2 (PMC10564882; doi:10.1038/s41598-023-43990-2)
Supplement: Supplementary file 1 — Supplementary Information 1. [file 41598_2023_43990_MOESM1_ESM.html]

Supplementary Methods & Results: Revisiting the relationship between illusory hand ownership induced by visuotactile synchrony and cardiac interoceptive accuracy


## Table of contents

- 1 Note
- 2 Load packages
- 3 Data preprocessing
- 4 Distributions of the variables
- 5 Bayesian statistical modeling
  - 5.1 Model discription
  - 5.2 Notation
  - 5.3 Model fitting
- 6 Posterior distributions of parameters and effect sizes
- 7 Additional analyses
  - 7.1 Trying other Priors
  - 7.2 Other items
- 8 About my enviroment

# Supplementary Methods & Results: Revisiting the relationship between illusory hand ownership induced by visuotactile synchrony and cardiac interoceptive accuracy

Author

Toyoki Yamagata, Kaito Ichikawa, Shogo Mizutori, Yusuke Haruki, & Kenji Ogawa

Published

October 5, 2023

# 1 Note

- This document was prepared by the 1st (T.Y.) and 3rd (S.M.) authors to provide the R programs used in our analyses and supplementary results.

# 2 Load packages

```
library(openxlsx)
library(tidyverse)
library(tidytext)
library(ggplot2)
theme_1 <- 
  theme_bw() +
  theme(
    panel.grid.major.x = element_blank(),
    panel.grid.minor.x = element_blank(),
    panel.grid.minor.y = element_blank(), 
    axis.title = element_text(size = 18), 
    axis.text = element_text(size = 18, colour = "black"), 
    axis.ticks = element_line(colour = "gray70")
    )
library(cmdstanr)
library(brms)
  options(mc.cores = parallel::detectCores())
  options(brms.backend = "cmdstanr")
  options(cmdstanr_write_stan_file_dir = ".")
  bayesplot::color_scheme_set("gray")
library(tidybayes)
```

# 3 Data preprocessing

- See Section 5.2 for the correspondence between variable names and their contents.

- Our data
- Open data

- The dataset generated by our experiment (`*_our`) will not be made publicly available, as informed consent for that was not obtained.
- The structure of our data is the same as that of the formatted open data from a previous study (see below).

- The raw data from Horváth et al. (2020) (`1-s2.0-S0010945220303312-mmc1.xlsx`) are available as supplementary data (Multimedia Component 1) at https://doi.org/10.1016/j.cortex.2020.08.026.

```
# load data
dat_open <- read.xlsx("./1-s2.0-S0010945220303312-mmc1.xlsx")

# data munging
excluded_list_open <- c(9, 35)

dat_open_1 <- dat_open %>%
  filter(!(participant %in% excluded_list_open)) %>%
  mutate(
    cia_25 = 1 - abs((Measured_25 - Reported_25) / Measured_25),
    cia_35 = 1 - abs((Measured_35 - Reported_35) / Measured_35),
    cia_50 = 1 - abs((Measured_50 - Reported_50) / Measured_50),
    x2 = (cia_25 + cia_35 + cia_50) / 3,
    x2_c = x2 - mean(x2)
    ) %>%
  gather(
    rubber_q_s1:rubber_q_as7,
    key = item_id_0,
    value = rating_0
    ) %>%
  transmute(
    sub_id = participant,
    x1 = if_else(grepl("a", item_id_0), 0, 1),
    Rating = factor(as.numeric(rating_0), ordered = T),
    item_id = str_replace_all(
      item_id_0, 
      c("rubber_q_s" = "y", "rubber_q_as" = "y")
      ),
    x2 = x2,
    x2_c =x2_c
    ) 

dat4fit_open <- dat_open_1 %>%
  spread(key = item_id, value = Rating) 

dat4fit_open_s <- dat4fit_open %>%
  filter(x1 == 1)
dat4fit_open_a <- dat4fit_open %>%
  filter(x1 == 0)
dat4fit_open_diff <- dat4fit_open_s %>%
  transmute(
    sub_id = sub_id,
    y3_diff = as.numeric(y3) - as.numeric(dat4fit_open_a$y3),
    x2 =x2,
    x2_c = x2 - mean(x2)
    )  
dat4fit_open$x2_c <- rep(dat4fit_open_diff$x2_c, each = 2)

dat_hist_open <- dat_open_1 %>%
  mutate(
    Condition = as.factor(x1),
    item_name = str_replace_all(item_id, c("y" = "Item "))
    )
```

# 4 Distributions of the variables

- Ratings on Likert-type questionnaire items
- Cardiac Interoceptive Accuracy (CIA)

- Our data
- Open data

```
gg_hist_rating_our <- 
  ggplot(
    data = dat_hist_our,
    mapping = aes(
      x = Rating,
      fill = Condition,
      colour = Condition
      )
    ) +
  facet_wrap(.~item_name, nrow = 2) +
  scale_y_continuous(name = "Count") +
  scale_colour_manual(
    values = c("#CC79A7", "#009E73"),
    guide = "none"
    ) + 
  scale_fill_manual(
    values=c("#CC79A7","#009E73"),
    labels = c("Asynchronous","Synchronous")
    ) +
  geom_bar(stat = "count", position = "identity", alpha = 0.3) +
  theme_1 +
  theme(
    legend.position = c(0.88, 0.25),
    legend.background = element_rect(colour = "black"),
    legend.key = element_blank(),
    legend.title = element_text(size = 14, colour = "black"),
    legend.text = element_text(size = 14, colour = "black"),
    strip.text = element_text(size = 14, colour = "black"),
    strip.text.x = element_text(size = 18)
    )

plot(gg_hist_rating_our)
```

Distributions of ratings in our data.

```
gg_hist_rating_open <- 
  ggplot(
    data = dat_hist_open,
    mapping = aes(
      x = Rating,
      fill = Condition,
      colour = Condition
      )
    ) +
  facet_wrap(.~item_name, nrow = 2) +
  scale_y_continuous(name = "Count") +
  scale_colour_manual(
    values = c("#CC79A7", "#009E73"),
    guide = "none"
    ) + 
  scale_fill_manual(
    values=c("#CC79A7","#009E73"),
    labels = c("Asynchronous","Synchronous")
    ) +
  geom_bar(stat = "count", position = "identity", alpha = 0.3) +
  theme_1 +
  theme(
    axis.text.x = element_text(size = 10, colour = "black"),
    legend.position = c(0.88, 0.25),
    legend.background = element_rect(colour = "black"),
    legend.key = element_blank(),
    legend.title = element_text(size = 14, colour = "black"),
    legend.text = element_text(size = 14, colour = "black"),
    strip.text = element_text(size = 14, colour = "black"),
    strip.text.x = element_text(size = 18)
    )

plot(gg_hist_rating_open)
```

Distributions of ratings in open data.

- Our data
- Open data

```
gg_hist_cia_our_0 <- dat_hist_our %>%
  select(sub_id, x2) %>%
  unique() %>%
  ggplot(aes(x = x2)) +
  geom_histogram(breaks = .05*(0:20), fill = "gray") +
  xlim(0, 1) +
  labs(x = expression(x[list(2,i)]), y = "count") +
  theme_1

gg_hist_cia_our <- gg_hist_cia_our_0 +
  annotate(
    "text",
    x = 0.5,
    hjust = .5,
    y = ggplot_build(gg_hist_cia_our_0)$layout$panel_params[[1]]$y.range[2],
    label = paste0(
      "M = ",
      round(mean(unique(dat_hist_our$x2)), 2),
      ", SD = ",
      round(sd(unique(dat_hist_our$x2)), 2)
      ),
    size = 8
    )

plot(gg_hist_cia_our)
```

Distribution of CIA in our data.

```
gg_hist_cia_open_0 <- dat_hist_open %>%
  select(sub_id, x2) %>%
  unique() %>%
  ggplot(aes(x = x2)) +
  geom_histogram(breaks = .05*(0:20), fill = "gray") +
  xlim(0, 1) +
  labs(x = expression(x[list(2,i)]), y = "count") +
  theme_1

gg_hist_cia_open <- gg_hist_cia_open_0 +
  annotate(
    "text",
    x = 0.5,
    hjust = .5,
    y = ggplot_build(gg_hist_cia_open_0)$layout$panel_params[[1]]$y.range[2],
    label = paste0(
      "M = ",
      round(mean(unique(dat_hist_open$x2)), 2),
      ", SD = ",
      round(sd(unique(dat_hist_open$x2)), 2)
      ),
    size = 8
    )

plot(gg_hist_cia_open)
```

Distribution of CIA in open data.

# 5 Bayesian statistical modeling

## 5.1 Model discription

- The below formulas describe the regression model we fit.
  - For the ordered-probit distribution, we followed the definition of Stan Functions Reference.

\[
\begin{gather}
y\_i \sim \text{OrderedProbit}(\mu\_{y,i}, \tau), \\
{y^\*}\_i = \mu\_{y,i} + e \\
\mu\_{y,i} = \delta\_{0,i} + \delta\_{1,i} x\_1, \\
\delta\_{0,i} = \beta\_2 x\_{2,i}, \\
\delta\_{1,i} = \beta\_1 + \beta\_3 x\_{2,i} + r\_i, \\
e \sim \text{Normal}(0,1), \\
r\_i \sim \text{Normal}(0,{\sigma\_r}^2).
\end{gather}
\]

## 5.2 Notation

- Table 1 summarizes the correspondence between the symbols in the model description and their contents.

Table 1: Notation of variables in our model.


| Symbol | Content |
| --- | --- |
| \(y\_i\) | Response to a Likert item |
| \({y^\*}\_i\) | Latent variable behind \(y\_i\) |
| \(\mu\_{y,i}\) | Expected value of \({y^\*}\_i\) |
| \(x\_1\) | Experimental condition (synchronous vs. asynchronous stimulation) |
| \(x\_{2,i}\) | Score of heart-beat tracking task |
| \(\delta\_{0,i}\) | \(\mu\_{y,i}\) at asynchronous stimulation (i.e., \(x\_1 = 0\)) |
| \(\delta\_{1,i}\) | Amount of change in \(\mu\_y\) due to synchronous stimulation (i.e., \(x\_1 = 1\)) |
| \(\beta\_1\) | Average effect of \(x\_1\) on \(y^\*\) at the population level |
| \(\beta\_2\) | Modulatory effect of \(x\_2\) on \(y^\*\) at baseline |
| \(\beta\_3\) | Modulatory effect of \(x\_2\) on \(\delta\_{1,i}\) |
| \(e\) | Normally distributed error in \(y^\*\) |
| \(r\_i\) | Normally distributed error in \(\delta\_1\) |
| \(\tau\) | Thresholds (nuisance parameter) |

## 5.3 Model fitting

- Model fittings were implemented using the Markov Chain Monte Carlo algorithm via the brms package.

- Our data
- Open data

```
formula_iho <-
   bf(
     y3 ~ x1*x2_c + (0 + x1 | sub_id), 
     family = cumulative("probit")
     )
prior_iho <- 
  prior(normal(0, 2.5), class = "b") +
  prior(normal(0, 2.5), class = "Intercept") 
prior_iho_exp <- prior_iho + 
  prior(exponential(1), class = "sd")
prior_iho_hC <- prior_iho + 
  prior(cauchy(0, 2.5), class = "sd")
prior_iho_hn <- prior_iho + 
  prior(normal(0, 2.5), class = "sd")

fit_our_0223 <- 
  brm(
    formula = formula_iho,
    data = dat4fit_our,
    prior = prior_iho,
    seed = 1, iter = 4000, warmup = 2000,
    file = "fit_our_0223",
    file_refit = "never",
    refresh = 2000
    )
fit_our_0223_hC <- 
  update(
    fit_our_0223,
    prior = prior_iho_hC,
    seed = 1,
    file = "fit_our_0223_hC",
    file_refit = "never",    
    refresh = 2000
    )
fit_our_0223_hn <- 
  update(
    fit_our_0223,
    prior = prior_iho_hn,
    seed = 1,
    file = "fit_our_0223_hn",
    file_refit = "never",
    refresh = 2000
    )
fit_our_0223_exp <- 
  update(
    fit_our_0223,
    prior = prior_iho_exp,
    seed = 1,
    file = "fit_our_0223_exp",
    file_refit = "never",
    refresh = 2000
    )
```

#### Used prior of \(\sigma\_r\)

- We considered the following four distributions as candidates for the prior of the variance parameter of the random slope:
  1. half-Cauchy with location = 0 and scale = 2.5
  2. half-normal with location = 0 and scale = 2.5
  3. *t* distribution with df = 3, location = 0, and scale = 2.5 (the default in brms)
  4. exponential with rate = 1
- While the results with each of the above four candidates are presented, we reported only the ones with the half-Cauchy in the article.

#### Confirm convergence: \(\hat R\)

- \(\hat R\)s of all parameters did not exceed 1.1.

- Half-Cauchy
- Half-normal
- *t*
- Exponential

```
bayesplot::mcmc_rhat(
  brms::rhat(fit_our_0223_hC)
  )
```

```
Warning: Dropped 1 NAs from 'new_rhat(rhat)'.
```

```
bayesplot::mcmc_rhat(
  brms::rhat(fit_our_0223_hn)
  )
```

```
Warning: Dropped 1 NAs from 'new_rhat(rhat)'.
```

```
bayesplot::mcmc_rhat(
  brms::rhat(fit_our_0223)
  )
```

```
Warning: Dropped 1 NAs from 'new_rhat(rhat)'.
```

```
bayesplot::mcmc_rhat(
  brms::rhat(fit_our_0223_exp)
  )
```

```
Warning: Dropped 1 NAs from 'new_rhat(rhat)'.
```

#### Confirm convergence: Traceplots

```
plot(
  fit_our_0223_hC, 
  variable = "^(b_x|sd_).*$", 
  regex = T, ask = F
  )
```

```
plot(
  fit_our_0223_hC, 
  variable = "^b_Intercept\\[\\d+\\]", 
  regex = T, ask = F
  )
```

#### Summary

- Half-Cauchy
- Half-normal
- *t*
- Exponential

```
summary(
  fit_our_0223_hC,
  priors = TRUE,
  prob = 0.95,  
  robust = TRUE
  )
```

```
 Family: cumulative 
  Links: mu = probit; disc = identity 
Formula: y3 ~ x1 * x2_c + (0 + x1 | sub_id) 
   Data: dat4fit_our (Number of observations: 100) 
  Draws: 4 chains, each with iter = 4000; warmup = 2000; thin = 1;
         total post-warmup draws = 8000

Priors: 
b ~ normal(0, 2.5)
Intercept ~ normal(0, 2.5)
<lower=0> sd ~ cauchy(0, 2.5)

Group-Level Effects: 
~sub_id (Number of levels: 50) 
       Estimate Est.Error l-95% CI u-95% CI Rhat Bulk_ESS Tail_ESS
sd(x1)     2.19      0.61     1.12     3.69 1.00     1801     2455

Population-Level Effects: 
             Estimate Est.Error l-95% CI u-95% CI Rhat Bulk_ESS Tail_ESS
Intercept[1]    -0.09      0.17    -0.43     0.26 1.00     7688     5959
Intercept[2]     0.90      0.19     0.52     1.26 1.00     8939     6440
Intercept[3]     1.53      0.24     1.07     2.03 1.00     5537     5228
Intercept[4]     2.02      0.32     1.43     2.73 1.00     4200     5019
Intercept[5]     4.50      0.96     2.94     6.80 1.00     2871     3539
x1               0.51      0.41    -0.35     1.33 1.00     3312     4424
x2_c             0.26      0.57    -0.89     1.41 1.00    12740     6317
x1:x2_c          0.33      1.33    -2.25     2.98 1.00     4077     5275

Family Specific Parameters: 
     Estimate Est.Error l-95% CI u-95% CI Rhat Bulk_ESS Tail_ESS
disc     1.00      0.00     1.00     1.00   NA       NA       NA

Draws were sampled using sample(hmc). For each parameter, Bulk_ESS
and Tail_ESS are effective sample size measures, and Rhat is the potential
scale reduction factor on split chains (at convergence, Rhat = 1).
```

```
summary(
  fit_our_0223_hn,
  priors = TRUE,
  prob = 0.95,  
  robust = TRUE
  )
```

```
 Family: cumulative 
  Links: mu = probit; disc = identity 
Formula: y3 ~ x1 * x2_c + (0 + x1 | sub_id) 
   Data: dat4fit_our (Number of observations: 100) 
  Draws: 4 chains, each with iter = 4000; warmup = 2000; thin = 1;
         total post-warmup draws = 8000

Priors: 
b ~ normal(0, 2.5)
Intercept ~ normal(0, 2.5)
<lower=0> sd ~ normal(0, 2.5)

Group-Level Effects: 
~sub_id (Number of levels: 50) 
       Estimate Est.Error l-95% CI u-95% CI Rhat Bulk_ESS Tail_ESS
sd(x1)     2.20      0.62     1.13     3.68 1.00     1603     2362

Population-Level Effects: 
             Estimate Est.Error l-95% CI u-95% CI Rhat Bulk_ESS Tail_ESS
Intercept[1]    -0.09      0.17    -0.43     0.26 1.00     8334     6124
Intercept[2]     0.89      0.19     0.52     1.27 1.00     9001     6114
Intercept[3]     1.53      0.25     1.07     2.03 1.00     5830     6121
Intercept[4]     2.02      0.33     1.45     2.73 1.00     3929     5097
Intercept[5]     4.54      0.96     2.98     6.79 1.00     2676     4026
x1               0.51      0.41    -0.35     1.35 1.00     2689     3775
x2_c             0.27      0.60    -0.89     1.43 1.00    11509     6209
x1:x2_c          0.33      1.29    -2.26     2.94 1.00     4259     5444

Family Specific Parameters: 
     Estimate Est.Error l-95% CI u-95% CI Rhat Bulk_ESS Tail_ESS
disc     1.00      0.00     1.00     1.00   NA       NA       NA

Draws were sampled using sample(hmc). For each parameter, Bulk_ESS
and Tail_ESS are effective sample size measures, and Rhat is the potential
scale reduction factor on split chains (at convergence, Rhat = 1).
```

```
summary(
  fit_our_0223,
  priors = TRUE,
  prob = 0.95,  
  robust = TRUE
  )
```

```
 Family: cumulative 
  Links: mu = probit; disc = identity 
Formula: y3 ~ x1 * x2_c + (0 + x1 | sub_id) 
   Data: dat4fit_our (Number of observations: 100) 
  Draws: 4 chains, each with iter = 4000; warmup = 2000; thin = 1;
         total post-warmup draws = 8000

Priors: 
b ~ normal(0, 2.5)
Intercept ~ normal(0, 2.5)
<lower=0> sd ~ student_t(3, 0, 2.5)

Group-Level Effects: 
~sub_id (Number of levels: 50) 
       Estimate Est.Error l-95% CI u-95% CI Rhat Bulk_ESS Tail_ESS
sd(x1)     2.19      0.59     1.19     3.61 1.00     1922     2954

Population-Level Effects: 
             Estimate Est.Error l-95% CI u-95% CI Rhat Bulk_ESS Tail_ESS
Intercept[1]    -0.09      0.17    -0.43     0.25 1.00     8722     6244
Intercept[2]     0.89      0.19     0.52     1.28 1.00    10691     6598
Intercept[3]     1.53      0.24     1.08     2.05 1.00     6785     6813
Intercept[4]     2.03      0.32     1.45     2.72 1.00     4882     6038
Intercept[5]     4.53      0.94     2.97     6.80 1.00     3524     5168
x1               0.51      0.40    -0.35     1.32 1.00     2895     4172
x2_c             0.26      0.59    -0.89     1.37 1.00    13089     6457
x1:x2_c          0.30      1.31    -2.29     2.99 1.00     4335     5467

Family Specific Parameters: 
     Estimate Est.Error l-95% CI u-95% CI Rhat Bulk_ESS Tail_ESS
disc     1.00      0.00     1.00     1.00   NA       NA       NA

Draws were sampled using sample(hmc). For each parameter, Bulk_ESS
and Tail_ESS are effective sample size measures, and Rhat is the potential
scale reduction factor on split chains (at convergence, Rhat = 1).
```

```
summary(
  fit_our_0223_exp,
  priors = TRUE,
  prob = 0.95,  
  robust = TRUE
  )
```

```
 Family: cumulative 
  Links: mu = probit; disc = identity 
Formula: y3 ~ x1 * x2_c + (0 + x1 | sub_id) 
   Data: dat4fit_our (Number of observations: 100) 
  Draws: 4 chains, each with iter = 4000; warmup = 2000; thin = 1;
         total post-warmup draws = 8000

Priors: 
b ~ normal(0, 2.5)
Intercept ~ normal(0, 2.5)
<lower=0> sd ~ exponential(1)

Group-Level Effects: 
~sub_id (Number of levels: 50) 
       Estimate Est.Error l-95% CI u-95% CI Rhat Bulk_ESS Tail_ESS
sd(x1)     1.96      0.56     0.96     3.33 1.00     1634     2478

Population-Level Effects: 
             Estimate Est.Error l-95% CI u-95% CI Rhat Bulk_ESS Tail_ESS
Intercept[1]    -0.07      0.18    -0.42     0.26 1.00     8877     5936
Intercept[2]     0.88      0.19     0.52     1.26 1.00     9764     6260
Intercept[3]     1.49      0.24     1.04     2.01 1.00     5786     6266
Intercept[4]     1.96      0.33     1.38     2.66 1.00     4032     5674
Intercept[5]     4.24      0.90     2.81     6.37 1.00     2725     3857
x1               0.52      0.39    -0.27     1.29 1.00     2836     3598
x2_c             0.24      0.59    -0.91     1.41 1.00    11520     6168
x1:x2_c          0.30      1.24    -2.21     2.90 1.00     4090     4460

Family Specific Parameters: 
     Estimate Est.Error l-95% CI u-95% CI Rhat Bulk_ESS Tail_ESS
disc     1.00      0.00     1.00     1.00   NA       NA       NA

Draws were sampled using sample(hmc). For each parameter, Bulk_ESS
and Tail_ESS are effective sample size measures, and Rhat is the potential
scale reduction factor on split chains (at convergence, Rhat = 1).
```

```
fit_open_0223 <- 
  update(
    fit_our_0223,
    newdata = dat4fit_open,
    seed = 1,
    file = "fit_open_0223",
    file_refit = "never",
    refresh = 2000
    )
fit_open_0223_hC <- 
  update(
    fit_our_0223_hC,
    newdata = dat4fit_open,
    seed = 1,
    file = "fit_open_0223_hC",
    file_refit = "never",
    refresh = 2000
    )
fit_open_0223_hn <- 
  update(
    fit_our_0223_hn,
    newdata = dat4fit_open,
    seed = 1,
    file = "fit_open_0223_hn",
    file_refit = "never",
    refresh = 2000
    )
fit_open_0223_exp <- 
  update(
    fit_our_0223_exp,
    newdata = dat4fit_open,
    seed = 1,
    control = list(adapt_delta = 0.99),
    file = "fit_open_0223_exp",
    file_refit = "never",
    refresh = 2000
    )
```

#### Used prior of \(\sigma\_r\)

- As in the case of our data.

#### Confirm convergence: \(\hat R\)

- \(\hat R\)s of all parameters did not exceed 1.1.

- Half-Cauchy
- Half-normal
- *t*
- Exponential

```
bayesplot::mcmc_rhat(
  brms::rhat(fit_open_0223_hC)
  )
```

```
Warning: Dropped 1 NAs from 'new_rhat(rhat)'.
```

```
bayesplot::mcmc_rhat(
  brms::rhat(fit_open_0223_hn)
  )
```

```
Warning: Dropped 1 NAs from 'new_rhat(rhat)'.
```

```
bayesplot::mcmc_rhat(
  brms::rhat(fit_open_0223)
  )
```

```
Warning: Dropped 1 NAs from 'new_rhat(rhat)'.
```

```
bayesplot::mcmc_rhat(
  brms::rhat(fit_open_0223_exp)
  )
```

```
Warning: Dropped 1 NAs from 'new_rhat(rhat)'.
```

#### Confirm convergence: Traceplots

```
plot(
  fit_open_0223_hC, 
  variable = "^(b_x|sd_).*$", 
  regex = T, ask = F
  )
```

```
plot(
  fit_open_0223_hC, 
  variable = "^b_Intercept\\[\\d+\\]", 
  regex = T, ask = F
  )
```

#### Summary

- Half-Cauchy
- Half-normal
- *t*
- Exponential

```
summary(
  fit_open_0223_hC,
  priors = TRUE,
  prob = 0.95,  
  robust = TRUE
  )
```

```
 Family: cumulative 
  Links: mu = probit; disc = identity 
Formula: y3 ~ x1 * x2_c + (0 + x1 | sub_id) 
   Data: dat4fit_open (Number of observations: 116) 
  Draws: 4 chains, each with iter = 4000; warmup = 2000; thin = 1;
         total post-warmup draws = 8000

Priors: 
b ~ normal(0, 2.5)
Intercept ~ normal(0, 2.5)
<lower=0> sd ~ cauchy(0, 2.5)

Group-Level Effects: 
~sub_id (Number of levels: 58) 
       Estimate Est.Error l-95% CI u-95% CI Rhat Bulk_ESS Tail_ESS
sd(x1)     1.17      0.43     0.28     2.10 1.01      819     1264

Population-Level Effects: 
              Estimate Est.Error l-95% CI u-95% CI Rhat Bulk_ESS Tail_ESS
Intercept[1]     -0.06      0.16    -0.37     0.26 1.00     6444     5981
Intercept[2]      0.28      0.16    -0.03     0.60 1.00     8602     6251
Intercept[3]      0.61      0.17     0.28     0.94 1.00    10000     6471
Intercept[4]      0.80      0.17     0.46     1.15 1.00    10091     6718
Intercept[5]      1.10      0.19     0.74     1.47 1.00     7732     7100
Intercept[6]      1.40      0.20     1.01     1.80 1.00     5267     6748
Intercept[7]      1.57      0.21     1.18     2.01 1.00     4008     6993
Intercept[8]      1.94      0.25     1.48     2.46 1.00     2395     5145
Intercept[9]      2.17      0.28     1.67     2.74 1.00     1970     4244
Intercept[10]     2.85      0.40     2.15     3.74 1.01     1284     3072
x1                1.75      0.31     1.20     2.43 1.00     2011     3396
x2_c             -0.60      0.46    -1.49     0.29 1.00     6919     6071
x1:x2_c           0.28      0.79    -1.29     1.85 1.00     4446     5059

Family Specific Parameters: 
     Estimate Est.Error l-95% CI u-95% CI Rhat Bulk_ESS Tail_ESS
disc     1.00      0.00     1.00     1.00   NA       NA       NA

Draws were sampled using sample(hmc). For each parameter, Bulk_ESS
and Tail_ESS are effective sample size measures, and Rhat is the potential
scale reduction factor on split chains (at convergence, Rhat = 1).
```

```
summary(
  fit_open_0223_hn,
  priors = TRUE,
  prob = 0.95,  
  robust = TRUE
  )
```

```
 Family: cumulative 
  Links: mu = probit; disc = identity 
Formula: y3 ~ x1 * x2_c + (0 + x1 | sub_id) 
   Data: dat4fit_open (Number of observations: 116) 
  Draws: 4 chains, each with iter = 4000; warmup = 2000; thin = 1;
         total post-warmup draws = 8000

Priors: 
b ~ normal(0, 2.5)
Intercept ~ normal(0, 2.5)
<lower=0> sd ~ normal(0, 2.5)

Group-Level Effects: 
~sub_id (Number of levels: 58) 
       Estimate Est.Error l-95% CI u-95% CI Rhat Bulk_ESS Tail_ESS
sd(x1)     1.20      0.44     0.26     2.15 1.00      897     1191

Population-Level Effects: 
              Estimate Est.Error l-95% CI u-95% CI Rhat Bulk_ESS Tail_ESS
Intercept[1]     -0.06      0.16    -0.37     0.25 1.00     7783     5865
Intercept[2]      0.28      0.16    -0.03     0.60 1.00    11045     6342
Intercept[3]      0.61      0.16     0.28     0.95 1.00    13187     6548
Intercept[4]      0.80      0.17     0.46     1.15 1.00    12050     6258
Intercept[5]      1.10      0.18     0.74     1.47 1.00     9247     6744
Intercept[6]      1.39      0.20     1.02     1.80 1.00     5479     6139
Intercept[7]      1.57      0.21     1.18     2.00 1.00     4173     6276
Intercept[8]      1.95      0.25     1.48     2.46 1.00     2533     5465
Intercept[9]      2.18      0.28     1.65     2.76 1.00     2094     4527
Intercept[10]     2.85      0.42     2.15     3.79 1.00     1485     3328
x1                1.75      0.31     1.19     2.47 1.00     1974     3001
x2_c             -0.60      0.47    -1.52     0.32 1.00     7747     5887
x1:x2_c           0.28      0.80    -1.35     1.87 1.00     4384     4911

Family Specific Parameters: 
     Estimate Est.Error l-95% CI u-95% CI Rhat Bulk_ESS Tail_ESS
disc     1.00      0.00     1.00     1.00   NA       NA       NA

Draws were sampled using sample(hmc). For each parameter, Bulk_ESS
and Tail_ESS are effective sample size measures, and Rhat is the potential
scale reduction factor on split chains (at convergence, Rhat = 1).
```

```
summary(
  fit_open_0223,
  priors = TRUE,
  prob = 0.95,  
  robust = TRUE
  )
```

```
 Family: cumulative 
  Links: mu = probit; disc = identity 
Formula: y3 ~ x1 * x2_c + (0 + x1 | sub_id) 
   Data: dat4fit_open (Number of observations: 116) 
  Draws: 4 chains, each with iter = 4000; warmup = 2000; thin = 1;
         total post-warmup draws = 8000

Priors: 
b ~ normal(0, 2.5)
Intercept ~ normal(0, 2.5)
<lower=0> sd ~ student_t(3, 0, 2.5)

Group-Level Effects: 
~sub_id (Number of levels: 58) 
       Estimate Est.Error l-95% CI u-95% CI Rhat Bulk_ESS Tail_ESS
sd(x1)     1.21      0.46     0.25     2.21 1.00      876     1026

Population-Level Effects: 
              Estimate Est.Error l-95% CI u-95% CI Rhat Bulk_ESS Tail_ESS
Intercept[1]     -0.06      0.16    -0.38     0.26 1.00     7334     5944
Intercept[2]      0.29      0.16    -0.04     0.60 1.00     9844     6688
Intercept[3]      0.60      0.17     0.28     0.94 1.00    10162     6810
Intercept[4]      0.80      0.18     0.46     1.15 1.00    10253     6510
Intercept[5]      1.09      0.19     0.73     1.47 1.00     7180     6874
Intercept[6]      1.39      0.21     1.01     1.81 1.00     4523     6429
Intercept[7]      1.57      0.22     1.16     2.02 1.00     3372     5638
Intercept[8]      1.94      0.26     1.46     2.49 1.00     2243     4406
Intercept[9]      2.17      0.28     1.64     2.78 1.00     1856     3762
Intercept[10]     2.86      0.42     2.13     3.87 1.00     1256     2782
x1                1.76      0.33     1.18     2.48 1.00     1777     2426
x2_c             -0.60      0.45    -1.52     0.31 1.00     8470     6346
x1:x2_c           0.29      0.80    -1.33     1.88 1.00     4139     4045

Family Specific Parameters: 
     Estimate Est.Error l-95% CI u-95% CI Rhat Bulk_ESS Tail_ESS
disc     1.00      0.00     1.00     1.00   NA       NA       NA

Draws were sampled using sample(hmc). For each parameter, Bulk_ESS
and Tail_ESS are effective sample size measures, and Rhat is the potential
scale reduction factor on split chains (at convergence, Rhat = 1).
```

```
summary(
  fit_open_0223_exp,
  priors = TRUE,
  prob = 0.95,  
  robust = TRUE
  )
```

```
 Family: cumulative 
  Links: mu = probit; disc = identity 
Formula: y3 ~ x1 * x2_c + (0 + x1 | sub_id) 
   Data: dat4fit_open (Number of observations: 116) 
  Draws: 4 chains, each with iter = 4000; warmup = 2000; thin = 1;
         total post-warmup draws = 8000

Priors: 
b ~ normal(0, 2.5)
Intercept ~ normal(0, 2.5)
<lower=0> sd ~ exponential(1)

Group-Level Effects: 
~sub_id (Number of levels: 58) 
       Estimate Est.Error l-95% CI u-95% CI Rhat Bulk_ESS Tail_ESS
sd(x1)     1.02      0.47     0.11     1.95 1.01      650     1157

Population-Level Effects: 
              Estimate Est.Error l-95% CI u-95% CI Rhat Bulk_ESS Tail_ESS
Intercept[1]     -0.05      0.16    -0.36     0.27 1.00     6578     5662
Intercept[2]      0.29      0.16    -0.03     0.61 1.00    10313     5457
Intercept[3]      0.60      0.17     0.28     0.95 1.00    12891     5992
Intercept[4]      0.80      0.17     0.46     1.15 1.00    12232     6583
Intercept[5]      1.08      0.18     0.73     1.45 1.00     8668     6574
Intercept[6]      1.37      0.21     0.99     1.78 1.00     4315     5977
Intercept[7]      1.54      0.22     1.14     1.98 1.00     3040     5659
Intercept[8]      1.90      0.26     1.42     2.42 1.00     1940     4557
Intercept[9]      2.11      0.28     1.59     2.69 1.00     1561     3875
Intercept[10]     2.74      0.40     2.04     3.61 1.00     1074     3183
x1                1.69      0.30     1.16     2.35 1.00     2003     3066
x2_c             -0.59      0.46    -1.50     0.30 1.00     8205     6268
x1:x2_c           0.27      0.77    -1.23     1.84 1.00     5187     5303

Family Specific Parameters: 
     Estimate Est.Error l-95% CI u-95% CI Rhat Bulk_ESS Tail_ESS
disc     1.00      0.00     1.00     1.00   NA       NA       NA

Draws were sampled using sample(hmc). For each parameter, Bulk_ESS
and Tail_ESS are effective sample size measures, and Rhat is the potential
scale reduction factor on split chains (at convergence, Rhat = 1).
```

# 6 Posterior distributions of parameters and effect sizes

- In the below block, a function to make the figure and to calculate the effect sizes is defined.

```
make_figure <- function(fit, data){
  # extract MCMC sample
  tidy_fit <- fit %>%
    spread_draws(
      b_x1, 
      r_sub_id[sub_id, term], 
      `b_x1:x2_c`, 
      sd_sub_id__x1
      ) %>%
    ungroup() %>%
    transmute(
      .draw = .draw,
      sub_id = sub_id,
      sub_id_chr = as.character(sub_id),
      sub_id_rev = -1 * sub_id,
      b1 = b_x1,
      r1 = r_sub_id,
      b3 = `b_x1:x2_c`
      )

  # calculating posterior median and 95%CrI of standardized beta_3
  std_b3 <- fit %>%
    spread_draws(
      `b_x1:x2_c`, 
      sd_sub_id__x1
      ) %>%
    ungroup() %>%
    transmute(std_b3 = `b_x1:x2_c` / sd_sub_id__x1) %>%
    median_qi()
  
  gg_std_b3 <- fit %>%
    spread_draws(
      `b_x1:x2_c`, 
      sd_sub_id__x1
      ) %>%
    ungroup() %>%
    transmute(std_b3 = `b_x1:x2_c` / sd_sub_id__x1) %>%
    ggplot() + 
    stat_halfeye(
      aes(x = std_b3), 
      point_interval = median_qi, 
      .width = c(0.95)
      ) + 
    coord_cartesian(xlim = c(-10, 10)) +
    xlab(expression(paste("Standardized ", beta[3])))
  
  # calculating posterior median and 95% HDPI of R2  
  R2 <- fit %>%
    spread_draws(
      `b_x1:x2_c`, 
      sd_sub_id__x1
      ) %>%
    ungroup() %>%
    mutate(
      b3 = `b_x1:x2_c`,
      sr = sd_sub_id__x1,
      ) %>%
    transmute(
      R2 = b3^2*var(data$x2) / (b3^2*var(data$x2) + sr^2)
      ) %>%
    median_hdi(.width = c(0.5, 0.95))  
  
   gg_R2 <- fit %>%
    spread_draws(
      `b_x1:x2_c`, 
      sd_sub_id__x1
      ) %>%
    ungroup() %>%
    mutate(
      b3 = `b_x1:x2_c`,
      sr = sd_sub_id__x1,
      ) %>%
    transmute(
      R2 = b3^2*var(data$x2) / (b3^2*var(data$x2) + sr^2)
      ) %>% 
    ggplot() + 
    stat_halfeye(
      aes(x = R2), 
      point_interval = median_hdi, 
      .width = c(0.5, 0.95)
      ) + 
    coord_cartesian(xlim = c(0, 0.5)) +     
    xlab(expression(R^2)) 

  # replicating y3_diff and x2
  y3_diff_x2_id_8000 <- 
    data.frame(
      y3_diff = rep(data$y3_diff, 8000),
      x2 = rep(data$x2, 8000),
      sub_id = rep(data$sub_id, 8000),
      .draw = sort(rep(1:8000, length(data$x2)))
      )

  # getting MCMC sample of delta_1
  dat4gg <- 
    inner_join(
      tidy_fit, 
      y3_diff_x2_id_8000, 
      by = c("sub_id", ".draw")
      ) %>%
    mutate(d1 = b1 + r1 + b3*x2) 
    
  # calculating 95%CrI of delta_1
  q95 <- 
    dat4gg %>%
    group_by(sub_id) %>%
    dplyr::summarise(
      q025 = quantile(d1, probs = 0.025, type = 7),
      q050 = quantile(d1, probs = 0.500, type = 7),
      q975 = quantile(d1, probs = 0.975, type = 7)
      ) %>%
    mutate(
      responder = q025 > 0
      )
  responder <- 
    group_by(q95, responder) %>%
    dplyr::summarise(number = n())
  
  # calculating rho
  rho <-
    dat4gg %>%
    group_by(.draw) %>%
    dplyr::summarise(rho = cor(d1, y3_diff)) %>%
    median_hdi(.width = c(0.5, 0.95))

  gg_rho <-
    dat4gg %>%
    group_by(.draw) %>%
    dplyr::summarise(rho = cor(d1, y3_diff)) %>%
    ggplot() + 
    stat_halfeye(
      aes(x = rho), 
      point_interval = median_hdi, 
      .width = c(0.5, 0.95)
      ) + 
    coord_cartesian(xlim = c(0, 1)) +    
    xlab(expression(rho)) 
  
  # making graph
  gg <- 
    ggplot(data = right_join(q95, dat4gg),
           aes(
             y = d1,
             x = x2,
             colour = responder
             )
           ) +
    ylab("Estimated IHO susceptibility") +
    stat_pointinterval(
      point_interval = median_qi,
      .width = c(.95),
      point_size = 3,
      alpha = 0.6
      ) + 
    scale_color_manual(values = c("#0072B2", "#E69F00")) +
    geom_hline(yintercept = 0, colour = "#CC79A7") +
    coord_cartesian(
      xlim = c(0, 1),
      ylim = c(-10, 10)
      ) +
    scale_x_continuous(
      name = "CIA",
      breaks = c(0, .25, .5, .75, 1)
      ) +
    theme_bw() +
    theme(
      panel.grid.major.x = element_blank(),
      panel.grid.minor.x = element_blank(),
      panel.grid.minor.y = element_blank(), 
      axis.title = element_text(size = 28),         
      axis.text = element_text(size = 20, colour = "black"), 
      axis.ticks = element_line(colour = "gray70"),
      legend.position = "none",
      plot.margin = margin(.75, .75, .75, .75, "cm")
      ) 
  
  # return
  return(list(
    gg = gg, responder = responder, 
    rho = rho, gg_rho = gg_rho,
    std_b3 = std_b3, gg_std_b3 = gg_std_b3,
    R2 = R2, gg_R2 = gg_R2
    ))
  }
```

- The plots visualizing the posterior distributions of the parameters and the quantities calculated from them are illustrated below.

- Our data
- Open data

#### \(x\_{2,i}\) and \(\delta\_{1,i}\)

```
# make figure
gg_color_our_hC <- make_figure(fit_our_0223_hC, dat4fit_our_diff)
```

```
Joining with `by = join_by(sub_id)`
```

```
gg_color_our_hn <- make_figure(fit_our_0223_hn, dat4fit_our_diff)
```

```
Joining with `by = join_by(sub_id)`
```

```
gg_color_our <- make_figure(fit_our_0223, dat4fit_our_diff)
```

```
Joining with `by = join_by(sub_id)`
```

```
gg_color_our_exp <- make_figure(fit_our_0223_exp, dat4fit_our_diff)
```

```
Joining with `by = join_by(sub_id)`
```

```
# plot
plot(gg_color_our_hC$gg)
```

\(x\_{2,i}\) and \(\delta\_{1,i}\) in our data

```
# save
ggsave(
  "d1i_our_022323.png", 
  plot = gg_color_our_hC$gg, device = "png", 
  width = 36, height = 18, 
  units = "cm", type = "cairo"
  )
```

```
Warning: Using ragg device as default. Ignoring `type` and `antialias` arguments
```

#### Frequency of “responders”

- The frequency of “responders,” or participants who unambiguously experienced IHO in response to our RHI induction, was estimated in each model.
  - In the case of the model with exponential 1, the estimated number of “responders” is lower than the others.

- Half-Cauchy
- Half-normal
- *t*
- Exponential

```
gg_color_our_hC$responder
```

```
# A tibble: 2 × 2
  responder number
  <lgl>      <int>
1 FALSE         38
2 TRUE          12
```

```
gg_color_our_hn$responder
```

```
# A tibble: 2 × 2
  responder number
  <lgl>      <int>
1 FALSE         37
2 TRUE          13
```

```
gg_color_our$responder
```

```
# A tibble: 2 × 2
  responder number
  <lgl>      <int>
1 FALSE         39
2 TRUE          11
```

```
gg_color_our_exp$responder
```

```
# A tibble: 2 × 2
  responder number
  <lgl>      <int>
1 FALSE         39
2 TRUE          11
```

#### \(\rho\)

- Half-Cauchy
- Half-normal
- *t*
- Exponential

```
gg_color_our_hC$rho
```

```
# A tibble: 2 × 6
    rho .lower .upper .width .point .interval
  <dbl>  <dbl>  <dbl>  <dbl> <chr>  <chr>    
1 0.674  0.638  0.718   0.5  median hdi      
2 0.674  0.540  0.787   0.95 median hdi
```

```
plot(gg_color_our_hC$gg_rho)
```

\(\rho\) in our data

```
gg_color_our_hn$rho
```

```
# A tibble: 2 × 6
    rho .lower .upper .width .point .interval
  <dbl>  <dbl>  <dbl>  <dbl> <chr>  <chr>    
1 0.677  0.645  0.726   0.5  median hdi      
2 0.677  0.541  0.790   0.95 median hdi
```

```
plot(gg_color_our_hn$gg_rho)
```

\(\rho\) in our data

```
gg_color_our$rho
```

```
# A tibble: 2 × 6
    rho .lower .upper .width .point .interval
  <dbl>  <dbl>  <dbl>  <dbl> <chr>  <chr>    
1 0.676  0.634  0.715   0.5  median hdi      
2 0.676  0.544  0.785   0.95 median hdi
```

```
plot(gg_color_our$gg_rho)
```

\(\rho\) in our data

```
gg_color_our_exp$rho
```

```
# A tibble: 2 × 6
    rho .lower .upper .width .point .interval
  <dbl>  <dbl>  <dbl>  <dbl> <chr>  <chr>    
1 0.665  0.632  0.718   0.5  median hdi      
2 0.665  0.506  0.784   0.95 median hdi
```

```
plot(gg_color_our_exp$gg_rho)
```

\(\rho\) in our data

#### \(\text{standardized }\beta\_3 = \frac{\beta\_3}{\sigma\_r}\)

- Half-Cauchy
- Half-normal
- *t*
- Exponential

```
gg_color_our_hC$std_b3
```

```
# A tibble: 1 × 6
  std_b3 .lower .upper .width .point .interval
   <dbl>  <dbl>  <dbl>  <dbl> <chr>  <chr>    
1  0.156  -1.09   1.40   0.95 median qi
```

```
plot(gg_color_our_hC$gg_std_b3)
```

standardized \(\beta\_3\) in our data

```
gg_color_our_hn$std_b3
```

```
# A tibble: 1 × 6
  std_b3 .lower .upper .width .point .interval
   <dbl>  <dbl>  <dbl>  <dbl> <chr>  <chr>    
1  0.155  -1.08   1.37   0.95 median qi
```

```
plot(gg_color_our_hn$gg_std_b3)
```

standardized \(\beta\_3\) in our data

```
gg_color_our$std_b3
```

```
# A tibble: 1 × 6
  std_b3 .lower .upper .width .point .interval
   <dbl>  <dbl>  <dbl>  <dbl> <chr>  <chr>    
1  0.142  -1.07   1.36   0.95 median qi
```

```
plot(gg_color_our$gg_std_b3)
```

standardized \(\beta\_3\) in our data

```
gg_color_our_exp$std_b3
```

```
# A tibble: 1 × 6
  std_b3 .lower .upper .width .point .interval
   <dbl>  <dbl>  <dbl>  <dbl> <chr>  <chr>    
1  0.164  -1.16   1.48   0.95 median qi
```

```
plot(gg_color_our_exp$gg_std_b3)
```

standardized \(\beta\_3\) in our data

#### \(R^2 = \frac{\beta\_3^2\text{var}(x\_{2,i})}{\beta\_3^2\text{var}(x\_{2,i}) + \sigma\_r^2}\)

- Half-Cauchy
- Half-normal
- *t*
- Exponential

```
gg_color_our_hC$R2
```

```
# A tibble: 2 × 6
      R2   .lower .upper .width .point .interval
   <dbl>    <dbl>  <dbl>  <dbl> <chr>  <chr>    
1 0.0118 1.94e-11 0.0118   0.5  median hdi      
2 0.0118 1.94e-11 0.0971   0.95 median hdi
```

```
plot(gg_color_our_hC$gg_R2)
```

\(R^2\) in our data

```
gg_color_our_hn$R2
```

```
# A tibble: 3 × 6
      R2       .lower .upper .width .point .interval
   <dbl>        <dbl>  <dbl>  <dbl> <chr>  <chr>    
1 0.0110 0.0000000225 0.0110   0.5  median hdi      
2 0.0110 0.0000000225 0.0944   0.95 median hdi      
3 0.0110 0.0998       0.103    0.95 median hdi
```

```
plot(gg_color_our_hn$gg_R2)
```

\(R^2\) in our data

```
gg_color_our$R2
```

```
# A tibble: 2 × 6
      R2   .lower .upper .width .point .interval
   <dbl>    <dbl>  <dbl>  <dbl> <chr>  <chr>    
1 0.0112 2.71e-10 0.0112   0.5  median hdi      
2 0.0112 2.71e-10 0.0967   0.95 median hdi
```

```
plot(gg_color_our$gg_R2)
```

\(R^2\) in our data

```
gg_color_our_exp$R2
```

```
# A tibble: 2 × 6
      R2   .lower .upper .width .point .interval
   <dbl>    <dbl>  <dbl>  <dbl> <chr>  <chr>    
1 0.0130 2.01e-12 0.0130   0.5  median hdi      
2 0.0130 2.01e-12 0.109    0.95 median hdi
```

```
plot(gg_color_our_exp$gg_R2)
```

\(R^2\) in our data

#### \(x\_{2,i}\) and \(\delta\_{1,i}\)

```
# make figure
gg_color_open_hC <- make_figure(fit_open_0223_hC, dat4fit_open_diff)
```

```
Joining with `by = join_by(sub_id)`
```

```
gg_color_open_hn <- make_figure(fit_open_0223_hn, dat4fit_open_diff)
```

```
Joining with `by = join_by(sub_id)`
```

```
gg_color_open <- make_figure(fit_open_0223, dat4fit_open_diff)
```

```
Joining with `by = join_by(sub_id)`
```

```
gg_color_open_exp <- make_figure(fit_open_0223_exp, dat4fit_open_diff)
```

```
Joining with `by = join_by(sub_id)`
```

```
# plot
plot(gg_color_open_hC$gg)
```

\(x\_{2,i}\) and \(\delta\_{1i}\) in Horváth et al. (2020)’s data

```
# save
ggsave(
  "d1i_open_022323.png", 
  plot = gg_color_open_hC$gg, device = "png", 
  width = 36, height = 18, 
  units = "cm", type = "cairo"
  )
```

```
Warning: Using ragg device as default. Ignoring `type` and `antialias` arguments
```

#### Frequency of “responders”

- The frequency of “responders,” or participants who unambiguously experienced IHO in response to our RHI induction, was estimated in each model.

- Half-Cauchy
- Half-normal
- *t*
- Exponential

```
gg_color_open_hC$responder
```

```
# A tibble: 2 × 2
  responder number
  <lgl>      <int>
1 FALSE         27
2 TRUE          31
```

```
gg_color_open_hn$responder
```

```
# A tibble: 2 × 2
  responder number
  <lgl>      <int>
1 FALSE         27
2 TRUE          31
```

```
gg_color_open$responder
```

```
# A tibble: 2 × 2
  responder number
  <lgl>      <int>
1 FALSE         27
2 TRUE          31
```

```
gg_color_open_exp$responder
```

```
# A tibble: 2 × 2
  responder number
  <lgl>      <int>
1 FALSE         26
2 TRUE          32
```

#### \(\rho\)

- Half-Cauchy
- Half-normal
- *t*
- Exponential

```
gg_color_open_hC$rho
```

```
# A tibble: 2 × 6
    rho .lower .upper .width .point .interval
  <dbl>  <dbl>  <dbl>  <dbl> <chr>  <chr>    
1 0.520  0.478  0.609   0.5  median hdi      
2 0.520  0.220  0.703   0.95 median hdi
```

```
plot(gg_color_open_hC$gg_rho)
```

\(\rho\) in open data

```
gg_color_open_hn$rho
```

```
# A tibble: 2 × 6
    rho .lower .upper .width .point .interval
  <dbl>  <dbl>  <dbl>  <dbl> <chr>  <chr>    
1 0.521  0.487  0.621   0.5  median hdi      
2 0.521  0.227  0.714   0.95 median hdi
```

```
plot(gg_color_open_hn$gg_rho)
```

\(\rho\) in open data

```
gg_color_open$rho
```

```
# A tibble: 2 × 6
    rho .lower .upper .width .point .interval
  <dbl>  <dbl>  <dbl>  <dbl> <chr>  <chr>    
1 0.525  0.492  0.627   0.5  median hdi      
2 0.525  0.222  0.712   0.95 median hdi
```

```
plot(gg_color_open$gg_rho)
```

\(\rho\) in open data

```
gg_color_open_exp$rho
```

```
# A tibble: 3 × 6
    rho .lower .upper .width .point .interval
  <dbl>  <dbl>  <dbl>  <dbl> <chr>  <chr>    
1 0.489 0.448   0.608   0.5  median hdi      
2 0.489 0.0654  0.116   0.95 median hdi      
3 0.489 0.151   0.692   0.95 median hdi
```

```
plot(gg_color_open_exp$gg_rho)
```

\(\rho\) in open data

#### \(\text{standardized }\beta\_3 = \frac{\beta\_3}{\sigma\_r}\)

- Half-Cauchy
- Half-normal
- *t*
- Exponential

```
gg_color_open_hC$std_b3
```

```
# A tibble: 1 × 6
  std_b3 .lower .upper .width .point .interval
   <dbl>  <dbl>  <dbl>  <dbl> <chr>  <chr>    
1  0.249  -1.24   2.37   0.95 median qi
```

```
plot(gg_color_open_hC$gg_std_b3)
```

standardized \(\beta\_3\) in Horváth et al. (2020)’s data

```
gg_color_open_hn$std_b3
```

```
# A tibble: 1 × 6
  std_b3 .lower .upper .width .point .interval
   <dbl>  <dbl>  <dbl>  <dbl> <chr>  <chr>    
1  0.240  -1.29   2.44   0.95 median qi
```

```
plot(gg_color_open_hn$gg_std_b3)
```

standardized \(\beta\_3\) in Horváth et al. (2020)’s data

```
gg_color_open$std_b3
```

```
# A tibble: 1 × 6
  std_b3 .lower .upper .width .point .interval
   <dbl>  <dbl>  <dbl>  <dbl> <chr>  <chr>    
1  0.242  -1.27   2.31   0.95 median qi
```

```
plot(gg_color_open$gg_std_b3)
```

standardized \(\beta\_3\) in Horváth et al. (2020)’s data

```
gg_color_open_exp$std_b3
```

```
# A tibble: 1 × 6
  std_b3 .lower .upper .width .point .interval
   <dbl>  <dbl>  <dbl>  <dbl> <chr>  <chr>    
1  0.286  -1.65   3.94   0.95 median qi
```

```
plot(gg_color_open_exp$gg_std_b3)
```

standardized \(\beta\_3\) in Horváth et al. (2020)’s data

#### \(R^2 = \frac{\beta\_3^2\text{var}(x\_{2,i})}{\beta\_3^2\text{var}(x\_{2,i}) + \sigma\_r^2}\)

- Half-Cauchy
- Half-normal
- *t*
- Exponential

```
gg_color_open_hC$R2
```

```
# A tibble: 2 × 6
      R2   .lower .upper .width .point .interval
   <dbl>    <dbl>  <dbl>  <dbl> <chr>  <chr>    
1 0.0227 2.69e-12 0.0227   0.5  median hdi      
2 0.0227 2.69e-12 0.247    0.95 median hdi
```

```
plot(gg_color_open_hC$gg_R2)
```

\(R^2\) in Horváth et al. (2020)’s data

```
gg_color_open_hn$R2
```

```
# A tibble: 2 × 6
      R2       .lower .upper .width .point .interval
   <dbl>        <dbl>  <dbl>  <dbl> <chr>  <chr>    
1 0.0234 0.0000000267 0.0234   0.5  median hdi      
2 0.0234 0.0000000267 0.259    0.95 median hdi
```

```
plot(gg_color_open_hn$gg_R2)
```

\(R^2\) in Horváth et al. (2020)’s data

```
gg_color_open$R2
```

```
# A tibble: 3 × 6
      R2   .lower .upper .width .point .interval
   <dbl>    <dbl>  <dbl>  <dbl> <chr>  <chr>    
1 0.0234 1.26e-13 0.0234   0.5  median hdi      
2 0.0234 1.26e-13 0.240    0.95 median hdi      
3 0.0234 2.50e- 1 0.275    0.95 median hdi
```

```
plot(gg_color_open$gg_R2)
```

\(R^2\) in Horváth et al. (2020)’s data

```
gg_color_open_exp$R2
```

```
# A tibble: 5 × 6
      R2   .lower .upper .width .point .interval
   <dbl>    <dbl>  <dbl>  <dbl> <chr>  <chr>    
1 0.0311 1.60e-10 0.0311   0.5  median hdi      
2 0.0311 1.60e-10 0.364    0.95 median hdi      
3 0.0311 3.74e- 1 0.421    0.95 median hdi      
4 0.0311 4.62e- 1 0.466    0.95 median hdi      
5 0.0311 9.65e- 1 0.990    0.95 median hdi
```

```
plot(gg_color_open_exp$gg_R2)
```

\(R^2\) in Horváth et al. (2020)’s data

# 7 Additional analyses

## 7.1 Trying other Priors

- We used a weakly informative prior for the coefficients in the analysis reported in our article.
- Since the result of Bayesian estimation, i.e. the posterior distribution, can be affected by the choice of prior, it is useful to try some other priors and see what differences they make.
- In this subsection, the results of estimation with two other priors are presented in each of the following panels: one is non-informative and the other is informative.

- Non-informative prior
- Informative prior

- For estimation with a non-informative prior, \(\text{Normal}(0, 10^2)\) was put for every coefficient parameter.
  - Since the error term in an ordered-probit regression has a unit variance, this distribution would be extraordinarily vague.
- The obtained posterior exhibited slightly larger uncertainty leaving its implication qualitatively unchanged.

```
formula_iho <-
   bf(
     y3 ~ x1*x2_c + (0 + x1 | sub_id), 
     family = cumulative("probit")
     )
prior_iho_hC_n010 <- 
  prior(normal(0, 10), class = "b") +
  prior(normal(0, 10), class = "Intercept") + 
  prior(cauchy(0, 2.5), class = "sd")

fit_our_hC_n010_0807 <- 
  brm(
    formula = formula_iho,
    data = dat4fit_our,
    prior = prior_iho_hC_n010,
    seed = 1, iter = 4000, warmup = 2000,
    file = "fit_our_hC_n010_0807",
    file_refit = "never",
    refresh = 2000
    )

fit_open_hC_n010_0807 <- 
  update(
    fit_our_hC_n010_0807,
    newdata = dat4fit_open,
    seed = 1,
    file = "fit_open_hC_n010_0807",
    file_refit = "never",
    refresh = 2000
    )
```

- Our data
- Open data

###### Confirm convergence: \(\hat R\)

- \(\hat R\)s of all parameters did not exceed 1.1.

```
bayesplot::mcmc_rhat(
  brms::rhat(fit_our_hC_n010_0807)
  )
```

```
Warning: Dropped 1 NAs from 'new_rhat(rhat)'.
```

###### Confirm convergence: Traceplots

```
plot(
  fit_our_hC_n010_0807, 
  variable = "^(b_x|sd_).*$", 
  regex = T, ask = F
  )
```

```
plot(
  fit_our_hC_n010_0807, 
  variable = "^b_Intercept\\[\\d+\\]", 
  regex = T, ask = F
  )
```

###### Summary

```
summary(
  fit_our_hC_n010_0807,
  priors = TRUE,
  prob = 0.95,  
  robust = TRUE
  )
```

```
 Family: cumulative 
  Links: mu = probit; disc = identity 
Formula: y3 ~ x1 * x2_c + (0 + x1 | sub_id) 
   Data: dat4fit_our (Number of observations: 100) 
  Draws: 4 chains, each with iter = 4000; warmup = 2000; thin = 1;
         total post-warmup draws = 8000

Priors: 
b ~ normal(0, 10)
Intercept ~ normal(0, 10)
<lower=0> sd ~ cauchy(0, 2.5)

Group-Level Effects: 
~sub_id (Number of levels: 50) 
       Estimate Est.Error l-95% CI u-95% CI Rhat Bulk_ESS Tail_ESS
sd(x1)     2.79      0.87     1.46     5.08 1.00     1282     2617

Population-Level Effects: 
             Estimate Est.Error l-95% CI u-95% CI Rhat Bulk_ESS Tail_ESS
Intercept[1]    -0.11      0.18    -0.46     0.24 1.00     7911     6410
Intercept[2]     0.93      0.20     0.54     1.32 1.00     9071     6970
Intercept[3]     1.64      0.27     1.15     2.19 1.00     4626     5691
Intercept[4]     2.22      0.37     1.58     3.05 1.00     3168     5416
Intercept[5]     5.74      1.55     3.41    10.05 1.00     1722     3691
x1               0.46      0.50    -0.73     1.44 1.00     1580     2137
x2_c             0.28      0.61    -0.92     1.47 1.00     8423     6405
x1:x2_c          0.70      1.84    -2.97     4.79 1.00     1753     2695

Family Specific Parameters: 
     Estimate Est.Error l-95% CI u-95% CI Rhat Bulk_ESS Tail_ESS
disc     1.00      0.00     1.00     1.00   NA       NA       NA

Draws were sampled using sample(hmc). For each parameter, Bulk_ESS
and Tail_ESS are effective sample size measures, and Rhat is the potential
scale reduction factor on split chains (at convergence, Rhat = 1).
```

###### Confirm convergence: \(\hat R\)

- \(\hat R\)s of all parameters did not exceed 1.1.

```
bayesplot::mcmc_rhat(
  brms::rhat(fit_open_hC_n010_0807)
  )
```

```
Warning: Dropped 1 NAs from 'new_rhat(rhat)'.
```

###### Confirm convergence: Traceplots

```
plot(
  fit_open_hC_n010_0807, 
  variable = "^(b_x|sd_).*$", 
  regex = T, ask = F
  )
```

```
plot(
  fit_open_hC_n010_0807, 
  variable = "^b_Intercept\\[\\d+\\]", 
  regex = T, ask = F
  )
```

###### Summary

```
summary(
  fit_open_hC_n010_0807,
  priors = TRUE,
  prob = 0.95,  
  robust = TRUE
  )
```

```
 Family: cumulative 
  Links: mu = probit; disc = identity 
Formula: y3 ~ x1 * x2_c + (0 + x1 | sub_id) 
   Data: dat4fit_open (Number of observations: 116) 
  Draws: 4 chains, each with iter = 4000; warmup = 2000; thin = 1;
         total post-warmup draws = 8000

Priors: 
b ~ normal(0, 10)
Intercept ~ normal(0, 10)
<lower=0> sd ~ cauchy(0, 2.5)

Group-Level Effects: 
~sub_id (Number of levels: 58) 
       Estimate Est.Error l-95% CI u-95% CI Rhat Bulk_ESS Tail_ESS
sd(x1)     1.28      0.43     0.39     2.24 1.01      845     1160

Population-Level Effects: 
              Estimate Est.Error l-95% CI u-95% CI Rhat Bulk_ESS Tail_ESS
Intercept[1]     -0.06      0.17    -0.37     0.26 1.00     7547     6437
Intercept[2]      0.29      0.17    -0.03     0.60 1.00    12163     6438
Intercept[3]      0.62      0.17     0.29     0.95 1.00    12594     6633
Intercept[4]      0.82      0.18     0.47     1.17 1.00    12384     6618
Intercept[5]      1.12      0.19     0.75     1.50 1.00     9734     5842
Intercept[6]      1.43      0.21     1.04     1.85 1.00     5956     6633
Intercept[7]      1.62      0.22     1.20     2.05 1.00     4058     5980
Intercept[8]      2.01      0.26     1.52     2.54 1.00     2401     4676
Intercept[9]      2.25      0.29     1.71     2.84 1.00     2046     4188
Intercept[10]     2.96      0.43     2.23     3.93 1.00     1374     3445
x1                1.82      0.34     1.22     2.54 1.00     2088     4052
x2_c             -0.64      0.47    -1.57     0.28 1.00     7965     6123
x1:x2_c           0.36      0.86    -1.43     2.08 1.00     3951     4245

Family Specific Parameters: 
     Estimate Est.Error l-95% CI u-95% CI Rhat Bulk_ESS Tail_ESS
disc     1.00      0.00     1.00     1.00   NA       NA       NA

Draws were sampled using sample(hmc). For each parameter, Bulk_ESS
and Tail_ESS are effective sample size measures, and Rhat is the potential
scale reduction factor on split chains (at convergence, Rhat = 1).
```

- For an informative prior, we put \(\text{Normal}(-2, 1)\) on the focal coefficient, \(\beta\_3\).
  - This choice is intended to reflect a hypothetical belief that might be formed from the result of Tsakiris et al. (2011).
  - They reported that the participants with higher levels of CIA tended to experience IHO less intensely, providing a statistic \(R^2 = .06\).
  - Since they did not provide the variance of IHO susceptibility, which is constructed in the conventional way, it is impossible to put a prior on the variance parameter \(\sigma\_r\) that is compatible with their result.
  - Nevertheless, we think \(\beta\_3 \sim \text{Normal}(-2, 1)\) roughly consistent with Tsakiris et al. (2011) because assuming \(\sigma\_r = 2.0\) (, which is close to the MEP of our analysis), \(\beta\_3 = 2.0\) implies \(R^2 \simeq .06\).
  - Use of this prior would reveal what rational belief the pair of the data and a prior that favors the result of Tsakiris et al. (2011) produces.
- In this estimation, \(\text{Normal}(0, 2.5^2)\), which is a weakly informative prior used in the primary analysis, is put on the rest of the coefficient parameters.
- The results showed that the existence of the mudulatory effect is uncertain even with a prior that assumes a certain size of it.
  - While \(\text{Normal}(-2, 1)\) has most of the mass in the negative domain, the posteriors ranged to the positive domain.

```
formula_iho <-
   bf(
     y3 ~ x1*x2_c + (0 + x1 | sub_id), 
     family = cumulative("probit")
     )
prior_iho_hC_n21 <- 
  prior(normal(0, 2.5), class = "b") +
  prior(normal(-2, 1), class = "b", coef = "x1:x2_c") +  
  prior(normal(0, 2.5), class = "Intercept") + 
  prior(cauchy(0, 2.5), class = "sd")

fit_our_hC_n21_0813 <- 
  brm(
    formula = formula_iho,
    data = dat4fit_our,
    prior = prior_iho_hC_n21,
    seed = 1, iter = 4000, warmup = 2000,
    file = "fit_our_hC_n21_0813",
    file_refit = "never",
    refresh = 2000
    )

fit_open_hC_n21_0813 <- 
  update(
    fit_our_hC_n21_0813,
    newdata = dat4fit_open,
    seed = 1,
    file = "fit_open_hC_n21_0813",
    file_refit = "never",
    refresh = 2000
    )
```

- Our data
- Open data

###### Confirm convergence: \(\hat R\)

- \(\hat R\)s of all parameters did not exceed 1.1.

```
bayesplot::mcmc_rhat(
  brms::rhat(fit_our_hC_n21_0813)
  )
```

```
Warning: Dropped 1 NAs from 'new_rhat(rhat)'.
```

###### Confirm convergence: Traceplots

```
plot(
  fit_our_hC_n21_0813, 
  variable = "^(b_x|sd_).*$", 
  regex = T, ask = F
  )
```

```
plot(
  fit_our_hC_n21_0813, 
  variable = "^b_Intercept\\[\\d+\\]", 
  regex = T, ask = F
  )
```

###### Summary

```
summary(
  fit_our_hC_n21_0813,
  priors = TRUE,
  prob = 0.95,  
  robust = TRUE
  )
```

```
 Family: cumulative 
  Links: mu = probit; disc = identity 
Formula: y3 ~ x1 * x2_c + (0 + x1 | sub_id) 
   Data: dat4fit_our (Number of observations: 100) 
  Draws: 4 chains, each with iter = 4000; warmup = 2000; thin = 1;
         total post-warmup draws = 8000

Priors: 
b ~ normal(0, 2.5)
b_x1:x2_c ~ normal(-2, 1)
Intercept ~ normal(0, 2.5)
<lower=0> sd ~ cauchy(0, 2.5)

Group-Level Effects: 
~sub_id (Number of levels: 50) 
       Estimate Est.Error l-95% CI u-95% CI Rhat Bulk_ESS Tail_ESS
sd(x1)     2.24      0.64     1.20     3.73 1.00     1493     2617

Population-Level Effects: 
             Estimate Est.Error l-95% CI u-95% CI Rhat Bulk_ESS Tail_ESS
Intercept[1]    -0.09      0.17    -0.43     0.24 1.00     7909     6785
Intercept[2]     0.89      0.19     0.54     1.27 1.00     8756     6967
Intercept[3]     1.54      0.25     1.07     2.04 1.00     5853     6776
Intercept[4]     2.04      0.32     1.46     2.74 1.00     3971     5127
Intercept[5]     4.52      0.97     3.00     6.74 1.00     2446     3639
x1               0.51      0.41    -0.39     1.33 1.00     2814     3817
x2_c             0.49      0.57    -0.65     1.63 1.00     9982     6075
x1:x2_c         -1.29      0.87    -2.95     0.35 1.00     5445     6239

Family Specific Parameters: 
     Estimate Est.Error l-95% CI u-95% CI Rhat Bulk_ESS Tail_ESS
disc     1.00      0.00     1.00     1.00   NA       NA       NA

Draws were sampled using sample(hmc). For each parameter, Bulk_ESS
and Tail_ESS are effective sample size measures, and Rhat is the potential
scale reduction factor on split chains (at convergence, Rhat = 1).
```

###### Confirm convergence: \(\hat R\)

- \(\hat R\)s of all parameters did not exceed 1.1.

```
bayesplot::mcmc_rhat(
  brms::rhat(fit_open_hC_n21_0813)
  )
```

```
Warning: Dropped 1 NAs from 'new_rhat(rhat)'.
```

###### Confirm convergence: Traceplots

```
plot(
  fit_open_hC_n21_0813, 
  variable = "^(b_x|sd_).*$", 
  regex = T, ask = F
  )
```

```
plot(
  fit_open_hC_n21_0813, 
  variable = "^b_Intercept\\[\\d+\\]", 
  regex = T, ask = F
  )
```

###### Summary

```
summary(
  fit_open_hC_n21_0813,
  priors = TRUE,
  prob = 0.95,  
  robust = TRUE
  )
```

```
 Family: cumulative 
  Links: mu = probit; disc = identity 
Formula: y3 ~ x1 * x2_c + (0 + x1 | sub_id) 
   Data: dat4fit_open (Number of observations: 116) 
  Draws: 4 chains, each with iter = 4000; warmup = 2000; thin = 1;
         total post-warmup draws = 8000

Priors: 
b ~ normal(0, 2.5)
b_x1:x2_c ~ normal(-2, 1)
Intercept ~ normal(0, 2.5)
<lower=0> sd ~ cauchy(0, 2.5)

Group-Level Effects: 
~sub_id (Number of levels: 58) 
       Estimate Est.Error l-95% CI u-95% CI Rhat Bulk_ESS Tail_ESS
sd(x1)     1.24      0.44     0.33     2.23 1.01      853     1042

Population-Level Effects: 
              Estimate Est.Error l-95% CI u-95% CI Rhat Bulk_ESS Tail_ESS
Intercept[1]     -0.07      0.16    -0.38     0.25 1.00     7061     6198
Intercept[2]      0.27      0.16    -0.04     0.60 1.00    10038     6577
Intercept[3]      0.60      0.17     0.27     0.93 1.00    11646     7408
Intercept[4]      0.79      0.18     0.46     1.15 1.00    11512     7052
Intercept[5]      1.09      0.19     0.74     1.46 1.00     9406     7109
Intercept[6]      1.39      0.20     1.00     1.80 1.00     6274     6639
Intercept[7]      1.57      0.22     1.17     2.00 1.00     4632     6485
Intercept[8]      1.95      0.25     1.49     2.48 1.00     2837     5069
Intercept[9]      2.18      0.28     1.67     2.75 1.00     2273     4766
Intercept[10]     2.87      0.41     2.17     3.81 1.00     1476     3071
x1                1.76      0.32     1.19     2.44 1.00     2066     2801
x2_c             -0.33      0.44    -1.19     0.52 1.00     8043     5868
x1:x2_c          -0.67      0.66    -2.07     0.60 1.00     4887     4855

Family Specific Parameters: 
     Estimate Est.Error l-95% CI u-95% CI Rhat Bulk_ESS Tail_ESS
disc     1.00      0.00     1.00     1.00   NA       NA       NA

Draws were sampled using sample(hmc). For each parameter, Bulk_ESS
and Tail_ESS are effective sample size measures, and Rhat is the potential
scale reduction factor on split chains (at convergence, Rhat = 1).
```

## 7.2 Other items

- In this subsection, the results obtained by applying the same model to the responses to the other Likert items than the third one are shown.
  - In summary, as with Item 3, no clear association with CIA was confirmed for the other items.
  - In addition, the results of Item 4, which is considered the most commonly used IHO measure after Item 3, can be interpreted in the same way as the results of Item 3.

- Item 1
- Item 2
- Item 4
- Item 5
- Item 6
- Item 7

```
formula_y1 <-
   bf(
     y1 ~ x1*x2_c + (0 + x1 | sub_id), 
     family = cumulative("probit")
     )
prior_otherItems <- 
  prior(normal(0, 2.5), class = "b") +
  prior(normal(0, 2.5), class = "Intercept") +
  prior(cauchy(0, 2.5), class = "sd")

fit_our_y1_hC_0716 <- 
  brm(
    formula = formula_y1,
    data = dat4fit_our,
    prior = prior_otherItems,
    seed = 1, iter = 4000, warmup = 2000,
    file = "fit_our_y1_0716",
    file_refit = "never",
    refresh = 2000
    )

fit_open_y1_hC_0807 <- 
  update(
    fit_our_y1_hC_0716,
    newdata = dat4fit_open,
    seed = 1,
    file = "fit_open_y1_0807",
    file_refit = "never",
    refresh = 2000
    )
```

- Our data
- Open data

###### Confirm convergence: \(\hat R\)

- \(\hat R\)s of all parameters did not exceed 1.1.

```
bayesplot::mcmc_rhat(
  brms::rhat(fit_our_y1_hC_0716)
  )
```

```
Warning: Dropped 1 NAs from 'new_rhat(rhat)'.
```

###### Confirm convergence: Traceplots

```
plot(
  fit_our_y1_hC_0716, 
  variable = "^(b_x|sd_).*$", 
  regex = T, ask = F
  )
```

```
plot(
  fit_our_y1_hC_0716, 
  variable = "^b_Intercept\\[\\d+\\]", 
  regex = T, ask = F
  )
```

###### Summary

```
summary(
  fit_our_y1_hC_0716,
  priors = TRUE,
  prob = 0.95,  
  robust = TRUE
  )
```

```
 Family: cumulative 
  Links: mu = probit; disc = identity 
Formula: y1 ~ x1 * x2_c + (0 + x1 | sub_id) 
   Data: dat4fit_our (Number of observations: 100) 
  Draws: 4 chains, each with iter = 4000; warmup = 2000; thin = 1;
         total post-warmup draws = 8000

Priors: 
b ~ normal(0, 2.5)
Intercept ~ normal(0, 2.5)
<lower=0> sd ~ cauchy(0, 2.5)

Group-Level Effects: 
~sub_id (Number of levels: 50) 
       Estimate Est.Error l-95% CI u-95% CI Rhat Bulk_ESS Tail_ESS
sd(x1)     1.44      0.53     0.35     2.66 1.00      836     1120

Population-Level Effects: 
             Estimate Est.Error l-95% CI u-95% CI Rhat Bulk_ESS Tail_ESS
Intercept[1]    -0.08      0.18    -0.43     0.26 1.00     5949     6331
Intercept[2]     0.68      0.18     0.33     1.04 1.00    11024     6695
Intercept[3]     0.93      0.19     0.57     1.31 1.00     9319     6929
Intercept[4]     1.05      0.19     0.67     1.45 1.00     7918     6462
Intercept[5]     1.88      0.29     1.35     2.51 1.00     2088     4629
Intercept[6]     3.11      0.60     2.15     4.54 1.00     1203     2620
x1               1.50      0.36     0.85     2.30 1.00     1981     2539
x2_c            -0.64      0.60    -1.84     0.49 1.00     9826     6250
x1:x2_c         -0.13      1.10    -2.39     1.97 1.00     4639     4298

Family Specific Parameters: 
     Estimate Est.Error l-95% CI u-95% CI Rhat Bulk_ESS Tail_ESS
disc     1.00      0.00     1.00     1.00   NA       NA       NA

Draws were sampled using sample(hmc). For each parameter, Bulk_ESS
and Tail_ESS are effective sample size measures, and Rhat is the potential
scale reduction factor on split chains (at convergence, Rhat = 1).
```

###### Confirm convergence: \(\hat R\)

- \(\hat R\)s of all parameters did not exceed 1.1.

```
bayesplot::mcmc_rhat(
  brms::rhat(fit_open_y1_hC_0807)
  )
```

```
Warning: Dropped 1 NAs from 'new_rhat(rhat)'.
```

###### Confirm convergence: Traceplots

```
plot(
  fit_open_y1_hC_0807, 
  variable = "^(b_x|sd_).*$", 
  regex = T, ask = F
  )
```

```
plot(
  fit_open_y1_hC_0807, 
  variable = "^b_Intercept\\[\\d+\\]", 
  regex = T, ask = F
  )
```

###### Summary

```
summary(
  fit_open_y1_hC_0807,
  priors = TRUE,
  prob = 0.95,  
  robust = TRUE
  )
```

```
 Family: cumulative 
  Links: mu = probit; disc = identity 
Formula: y1 ~ x1 * x2_c + (0 + x1 | sub_id) 
   Data: dat4fit_open (Number of observations: 116) 
  Draws: 4 chains, each with iter = 4000; warmup = 2000; thin = 1;
         total post-warmup draws = 8000

Priors: 
b ~ normal(0, 2.5)
Intercept ~ normal(0, 2.5)
<lower=0> sd ~ cauchy(0, 2.5)

Group-Level Effects: 
~sub_id (Number of levels: 58) 
       Estimate Est.Error l-95% CI u-95% CI Rhat Bulk_ESS Tail_ESS
sd(x1)     1.68      0.54     0.67     3.01 1.01      707     1204

Population-Level Effects: 
              Estimate Est.Error l-95% CI u-95% CI Rhat Bulk_ESS Tail_ESS
Intercept[1]     -0.11      0.16    -0.44     0.21 1.00     7209     6373
Intercept[2]      0.46      0.16     0.14     0.79 1.00     8656     6961
Intercept[3]      0.69      0.17     0.36     1.04 1.00     9229     6419
Intercept[4]      1.19      0.20     0.83     1.59 1.00     8063     6949
Intercept[5]      1.52      0.22     1.11     1.96 1.00     5569     6844
Intercept[6]      1.66      0.23     1.23     2.12 1.00     4574     6027
Intercept[7]      1.85      0.25     1.38     2.35 1.00     3719     5517
Intercept[8]      2.37      0.31     1.78     3.04 1.00     1950     3405
Intercept[9]      2.81      0.40     2.09     3.72 1.00     1237     2641
Intercept[10]     3.52      0.56     2.57     4.84 1.01      937     2092
x1                2.97      0.50     2.10     4.09 1.00     1024     2289
x2_c             -0.06      0.46    -0.95     0.86 1.00     8124     5378
x1:x2_c          -0.24      0.96    -2.18     1.70 1.00     3738     5328

Family Specific Parameters: 
     Estimate Est.Error l-95% CI u-95% CI Rhat Bulk_ESS Tail_ESS
disc     1.00      0.00     1.00     1.00   NA       NA       NA

Draws were sampled using sample(hmc). For each parameter, Bulk_ESS
and Tail_ESS are effective sample size measures, and Rhat is the potential
scale reduction factor on split chains (at convergence, Rhat = 1).
```

```
formula_y2 <-
   bf(
     y2 ~ x1*x2_c + (0 + x1 | sub_id), 
     family = cumulative("probit")
     )
prior_otherItems <- 
  prior(normal(0, 2.5), class = "b") +
  prior(normal(0, 2.5), class = "Intercept") +
  prior(cauchy(0, 2.5), class = "sd")

fit_our_y2_hC_0716 <- 
  brm(
    formula = formula_y2,
    data = dat4fit_our,
    prior = prior_otherItems,
    seed = 1, iter = 4000, warmup = 2000,
    file = "fit_our_y2_0716",
    file_refit = "never",
    refresh = 2000
    )

fit_open_y2_hC_0807 <- 
  update(
    fit_our_y2_hC_0716,
    newdata = dat4fit_open,
    seed = 1,
    file = "fit_open_y2_0807",
    file_refit = "never",
    refresh = 2000
    )
```

- Our data
- Open data

###### Confirm convergence: \(\hat R\)

- \(\hat R\)s of all parameters did not exceed 1.1.

```
bayesplot::mcmc_rhat(
  brms::rhat(fit_our_y2_hC_0716)
  )
```

```
Warning: Dropped 1 NAs from 'new_rhat(rhat)'.
```

###### Confirm convergence: Traceplots

```
plot(
  fit_our_y2_hC_0716, 
  variable = "^(b_x|sd_).*$", 
  regex = T, ask = F
  )
```

```
plot(
  fit_our_y2_hC_0716, 
  variable = "^b_Intercept\\[\\d+\\]", 
  regex = T, ask = F
  )
```

###### Summary

```
summary(
  fit_our_y2_hC_0716,
  priors = TRUE,
  prob = 0.95,  
  robust = TRUE
  )
```

```
Warning: There were 1 divergent transitions after
warmup. Increasing adapt_delta above 0.8 may help. See
http://mc-stan.org/misc/warnings.html#divergent-transitions-after-warmup
```

```
 Family: cumulative 
  Links: mu = probit; disc = identity 
Formula: y2 ~ x1 * x2_c + (0 + x1 | sub_id) 
   Data: dat4fit_our (Number of observations: 100) 
  Draws: 4 chains, each with iter = 4000; warmup = 2000; thin = 1;
         total post-warmup draws = 8000

Priors: 
b ~ normal(0, 2.5)
Intercept ~ normal(0, 2.5)
<lower=0> sd ~ cauchy(0, 2.5)

Group-Level Effects: 
~sub_id (Number of levels: 50) 
       Estimate Est.Error l-95% CI u-95% CI Rhat Bulk_ESS Tail_ESS
sd(x1)     1.07      0.45     0.15     2.03 1.01      901     1576

Population-Level Effects: 
             Estimate Est.Error l-95% CI u-95% CI Rhat Bulk_ESS Tail_ESS
Intercept[1]    -0.09      0.18    -0.42     0.25 1.00     6655     6393
Intercept[2]     0.61      0.18     0.26     0.96 1.00    12295     6125
Intercept[3]     1.12      0.20     0.72     1.53 1.00     7455     6070
Intercept[4]     1.39      0.22     0.97     1.83 1.00     5087     6622
Intercept[5]     2.60      0.44     1.90     3.72 1.00     1749     3367
Intercept[6]     3.93      0.79     2.67     5.93 1.00     1646     3464
x1               0.93      0.28     0.37     1.51 1.00     3728     4094
x2_c             0.39      0.56    -0.74     1.52 1.00     8561     5872
x1:x2_c         -0.26      0.96    -2.13     1.66 1.00     4486     4592

Family Specific Parameters: 
     Estimate Est.Error l-95% CI u-95% CI Rhat Bulk_ESS Tail_ESS
disc     1.00      0.00     1.00     1.00   NA       NA       NA

Draws were sampled using sample(hmc). For each parameter, Bulk_ESS
and Tail_ESS are effective sample size measures, and Rhat is the potential
scale reduction factor on split chains (at convergence, Rhat = 1).
```

###### Confirm convergence: \(\hat R\)

- \(\hat R\)s of all parameters did not exceed 1.1.

```
bayesplot::mcmc_rhat(
  brms::rhat(fit_open_y2_hC_0807)
  )
```

```
Warning: Dropped 1 NAs from 'new_rhat(rhat)'.
```

###### Confirm convergence: Traceplots

```
plot(
  fit_open_y2_hC_0807, 
  variable = "^(b_x|sd_).*$", 
  regex = T, ask = F
  )
```

```
plot(
  fit_open_y2_hC_0807, 
  variable = "^b_Intercept\\[\\d+\\]", 
  regex = T, ask = F
  )
```

###### Summary

```
summary(
  fit_open_y2_hC_0807,
  priors = TRUE,
  prob = 0.95,  
  robust = TRUE
  )
```

```
 Family: cumulative 
  Links: mu = probit; disc = identity 
Formula: y2 ~ x1 * x2_c + (0 + x1 | sub_id) 
   Data: dat4fit_open (Number of observations: 116) 
  Draws: 4 chains, each with iter = 4000; warmup = 2000; thin = 1;
         total post-warmup draws = 8000

Priors: 
b ~ normal(0, 2.5)
Intercept ~ normal(0, 2.5)
<lower=0> sd ~ cauchy(0, 2.5)

Group-Level Effects: 
~sub_id (Number of levels: 58) 
       Estimate Est.Error l-95% CI u-95% CI Rhat Bulk_ESS Tail_ESS
sd(x1)     1.73      0.46     0.90     2.85 1.01      985     1977

Population-Level Effects: 
              Estimate Est.Error l-95% CI u-95% CI Rhat Bulk_ESS Tail_ESS
Intercept[1]     -0.27      0.16    -0.59     0.06 1.00     6636     5848
Intercept[2]      0.19      0.16    -0.13     0.51 1.00     8728     6531
Intercept[3]      0.79      0.17     0.44     1.14 1.00     9133     6819
Intercept[4]      1.15      0.19     0.79     1.54 1.00     8454     6739
Intercept[5]      1.58      0.23     1.14     2.05 1.00     4680     5517
Intercept[6]      1.93      0.28     1.42     2.53 1.00     3228     5551
Intercept[7]      2.43      0.35     1.79     3.19 1.00     2169     4458
Intercept[8]      2.69      0.38     2.00     3.53 1.00     1943     3491
Intercept[9]      3.24      0.45     2.42     4.24 1.00     1717     3186
Intercept[10]     4.04      0.63     2.94     5.43 1.00     1441     2874
x1                2.19      0.41     1.46     3.10 1.00     1667     2862
x2_c              0.27      0.47    -0.62     1.15 1.00     9848     6043
x1:x2_c           0.02      0.93    -1.80     1.90 1.00     3690     4688

Family Specific Parameters: 
     Estimate Est.Error l-95% CI u-95% CI Rhat Bulk_ESS Tail_ESS
disc     1.00      0.00     1.00     1.00   NA       NA       NA

Draws were sampled using sample(hmc). For each parameter, Bulk_ESS
and Tail_ESS are effective sample size measures, and Rhat is the potential
scale reduction factor on split chains (at convergence, Rhat = 1).
```

```
formula_y4 <-
   bf(
     y4 ~ x1*x2_c + (0 + x1 | sub_id), 
     family = cumulative("probit")
     )
prior_otherItems <- 
  prior(normal(0, 2.5), class = "b") +
  prior(normal(0, 2.5), class = "Intercept") +
  prior(cauchy(0, 2.5), class = "sd")

fit_our_y4_hC_0716 <- 
  brm(
    formula = formula_y4,
    data = dat4fit_our,
    prior = prior_otherItems,
    seed = 1, iter = 4000, warmup = 2000,
    file = "fit_our_y4_0716",
    file_refit = "never",
    refresh = 2000
    )

fit_open_y4_hC_0807 <- 
  update(
    fit_our_y4_hC_0716,
    newdata = dat4fit_open,
    seed = 1,
    file = "fit_open_y4_0807",
    file_refit = "never",
    refresh = 2000
    )
```

- Our data
- Open data

###### Confirm convergence: \(\hat R\)

- \(\hat R\)s of all parameters did not exceed 1.1.

```
bayesplot::mcmc_rhat(
  brms::rhat(fit_our_y4_hC_0716)
  )
```

```
Warning: Dropped 1 NAs from 'new_rhat(rhat)'.
```

###### Confirm convergence: Traceplots

```
plot(
  fit_our_y4_hC_0716, 
  variable = "^(b_x|sd_).*$", 
  regex = T, ask = F
  )
```

```
plot(
  fit_our_y4_hC_0716, 
  variable = "^b_Intercept\\[\\d+\\]", 
  regex = T, ask = F
  )
```

###### Summary

```
summary(
  fit_our_y4_hC_0716,
  priors = TRUE,
  prob = 0.95,  
  robust = TRUE
  )
```

```
 Family: cumulative 
  Links: mu = probit; disc = identity 
Formula: y4 ~ x1 * x2_c + (0 + x1 | sub_id) 
   Data: dat4fit_our (Number of observations: 100) 
  Draws: 4 chains, each with iter = 4000; warmup = 2000; thin = 1;
         total post-warmup draws = 8000

Priors: 
b ~ normal(0, 2.5)
Intercept ~ normal(0, 2.5)
<lower=0> sd ~ cauchy(0, 2.5)

Group-Level Effects: 
~sub_id (Number of levels: 50) 
       Estimate Est.Error l-95% CI u-95% CI Rhat Bulk_ESS Tail_ESS
sd(x1)     1.49      0.50     0.49     2.55 1.01      946      915

Population-Level Effects: 
             Estimate Est.Error l-95% CI u-95% CI Rhat Bulk_ESS Tail_ESS
Intercept[1]    -0.10      0.18    -0.44     0.25 1.00     7038     5492
Intercept[2]     0.74      0.18     0.39     1.11 1.00    13349     6067
Intercept[3]     1.22      0.21     0.81     1.65 1.00     6084     6560
Intercept[4]     1.73      0.27     1.22     2.28 1.00     3263     4416
Intercept[5]     3.26      0.62     2.21     4.69 1.00     1785     2157
Intercept[6]     4.26      0.90     2.79     6.38 1.01     1700     2506
x1               0.61      0.32    -0.03     1.28 1.00     3350     3332
x2_c             0.22      0.59    -0.97     1.38 1.00     9238     6248
x1:x2_c          0.14      1.11    -2.08     2.35 1.00     4397     4804

Family Specific Parameters: 
     Estimate Est.Error l-95% CI u-95% CI Rhat Bulk_ESS Tail_ESS
disc     1.00      0.00     1.00     1.00   NA       NA       NA

Draws were sampled using sample(hmc). For each parameter, Bulk_ESS
and Tail_ESS are effective sample size measures, and Rhat is the potential
scale reduction factor on split chains (at convergence, Rhat = 1).
```

###### Confirm convergence: \(\hat R\)

- \(\hat R\)s of all parameters did not exceed 1.1.

```
bayesplot::mcmc_rhat(
  brms::rhat(fit_open_y4_hC_0807)
  )
```

```
Warning: Dropped 1 NAs from 'new_rhat(rhat)'.
```

###### Confirm convergence: Traceplots

```
plot(
  fit_open_y4_hC_0807, 
  variable = "^(b_x|sd_).*$", 
  regex = T, ask = F
  )
```

```
plot(
  fit_open_y4_hC_0807, 
  variable = "^b_Intercept\\[\\d+\\]", 
  regex = T, ask = F
  )
```

###### Summary

```
summary(
  fit_open_y4_hC_0807,
  priors = TRUE,
  prob = 0.95,  
  robust = TRUE
  )
```

```
Warning: There were 17 divergent transitions after
warmup. Increasing adapt_delta above 0.8 may help. See
http://mc-stan.org/misc/warnings.html#divergent-transitions-after-warmup
```

```
 Family: cumulative 
  Links: mu = probit; disc = identity 
Formula: y4 ~ x1 * x2_c + (0 + x1 | sub_id) 
   Data: dat4fit_open (Number of observations: 116) 
  Draws: 4 chains, each with iter = 4000; warmup = 2000; thin = 1;
         total post-warmup draws = 8000

Priors: 
b ~ normal(0, 2.5)
Intercept ~ normal(0, 2.5)
<lower=0> sd ~ cauchy(0, 2.5)

Group-Level Effects: 
~sub_id (Number of levels: 58) 
       Estimate Est.Error l-95% CI u-95% CI Rhat Bulk_ESS Tail_ESS
sd(x1)     0.64      0.39     0.04     1.38 1.00      949     2481

Population-Level Effects: 
              Estimate Est.Error l-95% CI u-95% CI Rhat Bulk_ESS Tail_ESS
Intercept[1]     -0.15      0.16    -0.46     0.17 1.00     6793     5471
Intercept[2]      0.34      0.16     0.03     0.66 1.00    10036     5536
Intercept[3]      0.54      0.16     0.23     0.86 1.00    10965     5946
Intercept[4]      0.85      0.18     0.52     1.19 1.00     9644     6492
Intercept[5]      1.05      0.18     0.70     1.41 1.00     8270     6067
Intercept[6]      1.27      0.19     0.91     1.66 1.00     6226     6078
Intercept[7]      1.51      0.20     1.13     1.93 1.00     4678     5871
Intercept[8]      1.76      0.22     1.34     2.21 1.00     3351     4378
Intercept[9]      2.14      0.26     1.66     2.70 1.00     2422     3955
Intercept[10]     2.59      0.32     2.03     3.28 1.00     1949     3698
x1                1.39      0.24     0.95     1.89 1.00     3486     2965
x2_c             -0.53      0.46    -1.41     0.37 1.00     6774     5821
x1:x2_c           0.46      0.70    -0.91     1.85 1.00     5823     4243

Family Specific Parameters: 
     Estimate Est.Error l-95% CI u-95% CI Rhat Bulk_ESS Tail_ESS
disc     1.00      0.00     1.00     1.00   NA       NA       NA

Draws were sampled using sample(hmc). For each parameter, Bulk_ESS
and Tail_ESS are effective sample size measures, and Rhat is the potential
scale reduction factor on split chains (at convergence, Rhat = 1).
```

```
formula_y5 <-
   bf(
     y5 ~ x1*x2_c + (0 + x1 | sub_id), 
     family = cumulative("probit")
     )
prior_otherItems <- 
  prior(normal(0, 2.5), class = "b") +
  prior(normal(0, 2.5), class = "Intercept") +
  prior(cauchy(0, 2.5), class = "sd")

fit_our_y5_hC_0716 <- 
  brm(
    formula = formula_y5,
    data = dat4fit_our,
    prior = prior_otherItems,
    seed = 1, iter = 4000, warmup = 2000,
    file = "fit_our_y5_0716",
    file_refit = "never",
    refresh = 2000
    )

fit_open_y5_hC_0807 <- 
  update(
    fit_our_y5_hC_0716,
    newdata = dat4fit_open,
    seed = 1,
    file = "fit_open_y5_0807",
    file_refit = "never",
    refresh = 2000
    )
```

- Our data
- Open data

###### Confirm convergence: \(\hat R\)

- \(\hat R\)s of all parameters did not exceed 1.1.

```
bayesplot::mcmc_rhat(
  brms::rhat(fit_our_y5_hC_0716)
  )
```

```
Warning: Dropped 1 NAs from 'new_rhat(rhat)'.
```

###### Confirm convergence: Traceplots

```
plot(
  fit_our_y5_hC_0716, 
  variable = "^(b_x|sd_).*$", 
  regex = T, ask = F
  )
```

```
plot(
  fit_our_y5_hC_0716, 
  variable = "^b_Intercept\\[\\d+\\]", 
  regex = T, ask = F
  )
```

###### Summary

```
summary(
  fit_our_y5_hC_0716,
  priors = TRUE,
  prob = 0.95,  
  robust = TRUE
  )
```

```
Warning: There were 9 divergent transitions after
warmup. Increasing adapt_delta above 0.8 may help. See
http://mc-stan.org/misc/warnings.html#divergent-transitions-after-warmup
```

```
 Family: cumulative 
  Links: mu = probit; disc = identity 
Formula: y5 ~ x1 * x2_c + (0 + x1 | sub_id) 
   Data: dat4fit_our (Number of observations: 100) 
  Draws: 4 chains, each with iter = 4000; warmup = 2000; thin = 1;
         total post-warmup draws = 8000

Priors: 
b ~ normal(0, 2.5)
Intercept ~ normal(0, 2.5)
<lower=0> sd ~ cauchy(0, 2.5)

Group-Level Effects: 
~sub_id (Number of levels: 50) 
       Estimate Est.Error l-95% CI u-95% CI Rhat Bulk_ESS Tail_ESS
sd(x1)     0.46      0.36     0.02     1.25 1.00     1175     2627

Population-Level Effects: 
             Estimate Est.Error l-95% CI u-95% CI Rhat Bulk_ESS Tail_ESS
Intercept[1]    -0.32      0.17    -0.65     0.01 1.00     7427     4475
Intercept[2]     0.20      0.17    -0.13     0.53 1.00    11192     5647
Intercept[3]     0.70      0.18     0.35     1.06 1.00     9271     5243
Intercept[4]     0.95      0.19     0.57     1.34 1.00     8546     5774
Intercept[5]     1.26      0.20     0.87     1.69 1.00     7174     4836
Intercept[6]     2.47      0.42     1.78     3.40 1.00     8014     5723
x1              -0.16      0.23    -0.66     0.30 1.00     4896     3440
x2_c             0.51      0.58    -0.64     1.64 1.00     5810     5333
x1:x2_c         -0.56      0.87    -2.26     1.14 1.00     4945     5084

Family Specific Parameters: 
     Estimate Est.Error l-95% CI u-95% CI Rhat Bulk_ESS Tail_ESS
disc     1.00      0.00     1.00     1.00   NA       NA       NA

Draws were sampled using sample(hmc). For each parameter, Bulk_ESS
and Tail_ESS are effective sample size measures, and Rhat is the potential
scale reduction factor on split chains (at convergence, Rhat = 1).
```

###### Confirm convergence: \(\hat R\)

- \(\hat R\)s of all parameters did not exceed 1.1.

```
bayesplot::mcmc_rhat(
  brms::rhat(fit_open_y5_hC_0807)
  )
```

```
Warning: Dropped 1 NAs from 'new_rhat(rhat)'.
```

###### Confirm convergence: Traceplots

```
plot(
  fit_open_y5_hC_0807, 
  variable = "^(b_x|sd_).*$", 
  regex = T, ask = F
  )
```

```
plot(
  fit_open_y5_hC_0807, 
  variable = "^b_Intercept\\[\\d+\\]", 
  regex = T, ask = F
  )
```

###### Summary

```
summary(
  fit_open_y5_hC_0807,
  priors = TRUE,
  prob = 0.95,  
  robust = TRUE
  )
```

```
Warning: There were 30 divergent transitions after
warmup. Increasing adapt_delta above 0.8 may help. See
http://mc-stan.org/misc/warnings.html#divergent-transitions-after-warmup
```

```
 Family: cumulative 
  Links: mu = probit; disc = identity 
Formula: y5 ~ x1 * x2_c + (0 + x1 | sub_id) 
   Data: dat4fit_open (Number of observations: 116) 
  Draws: 4 chains, each with iter = 4000; warmup = 2000; thin = 1;
         total post-warmup draws = 8000

Priors: 
b ~ normal(0, 2.5)
Intercept ~ normal(0, 2.5)
<lower=0> sd ~ cauchy(0, 2.5)

Group-Level Effects: 
~sub_id (Number of levels: 58) 
       Estimate Est.Error l-95% CI u-95% CI Rhat Bulk_ESS Tail_ESS
sd(x1)     0.49      0.36     0.02     1.28 1.00      661      497

Population-Level Effects: 
              Estimate Est.Error l-95% CI u-95% CI Rhat Bulk_ESS Tail_ESS
Intercept[1]     -0.20      0.16    -0.52     0.11 1.00     1995      834
Intercept[2]      0.13      0.15    -0.17     0.43 1.00     5680     3803
Intercept[3]      0.54      0.16     0.23     0.85 1.00     6329     5487
Intercept[4]      0.69      0.16     0.38     1.01 1.00     6169     5141
Intercept[5]      0.93      0.17     0.61     1.27 1.00     6014     5319
Intercept[6]      1.08      0.18     0.74     1.44 1.00     5212     5556
Intercept[7]      1.24      0.19     0.88     1.62 1.00     4303     3988
Intercept[8]      1.41      0.20     1.04     1.83 1.00     3463     3811
Intercept[9]      1.68      0.22     1.27     2.15 1.00     2528     2917
Intercept[10]     2.03      0.24     1.57     2.56 1.00     1773     1526
x1                0.62      0.21     0.17     1.05 1.00     1488      560
x2_c              0.28      0.47    -0.62     1.20 1.00     5296     5224
x1:x2_c          -1.02      0.70    -2.36     0.36 1.00     2849     2948

Family Specific Parameters: 
     Estimate Est.Error l-95% CI u-95% CI Rhat Bulk_ESS Tail_ESS
disc     1.00      0.00     1.00     1.00   NA       NA       NA

Draws were sampled using sample(hmc). For each parameter, Bulk_ESS
and Tail_ESS are effective sample size measures, and Rhat is the potential
scale reduction factor on split chains (at convergence, Rhat = 1).
```

```
formula_y6 <-
   bf(
     y6 ~ x1*x2_c + (0 + x1 | sub_id), 
     family = cumulative("probit")
     )
prior_otherItems <- 
  prior(normal(0, 2.5), class = "b") +
  prior(normal(0, 2.5), class = "Intercept") +
  prior(cauchy(0, 2.5), class = "sd")

fit_our_y6_hC_0716 <- 
  brm(
    formula = formula_y6,
    data = dat4fit_our,
    prior = prior_otherItems,
    seed = 1, iter = 4000, warmup = 2000,
    file = "fit_our_y6_0716",
    file_refit = "never",
    refresh = 2000
    )

fit_open_y6_hC_0807 <- 
  update(
    fit_our_y6_hC_0716,
    newdata = dat4fit_open,
    seed = 1,
    file = "fit_open_y6_0807",
    file_refit = "never",
    refresh = 2000
    )
```

- Our data
- Open data

###### Confirm convergence: \(\hat R\)

- \(\hat R\)s of all parameters did not exceed 1.1.

```
bayesplot::mcmc_rhat(
  brms::rhat(fit_our_y6_hC_0716)
  )
```

```
Warning: Dropped 1 NAs from 'new_rhat(rhat)'.
```

###### Confirm convergence: Traceplots

```
plot(
  fit_our_y6_hC_0716, 
  variable = "^(b_x|sd_).*$", 
  regex = T, ask = F
  )
```

```
plot(
  fit_our_y6_hC_0716, 
  variable = "^b_Intercept\\[\\d+\\]", 
  regex = T, ask = F
  )
```

###### Summary

```
summary(
  fit_our_y6_hC_0716,
  priors = TRUE,
  prob = 0.95,  
  robust = TRUE
  )
```

```
Warning: There were 6 divergent transitions after
warmup. Increasing adapt_delta above 0.8 may help. See
http://mc-stan.org/misc/warnings.html#divergent-transitions-after-warmup
```

```
 Family: cumulative 
  Links: mu = probit; disc = identity 
Formula: y6 ~ x1 * x2_c + (0 + x1 | sub_id) 
   Data: dat4fit_our (Number of observations: 100) 
  Draws: 4 chains, each with iter = 4000; warmup = 2000; thin = 1;
         total post-warmup draws = 8000

Priors: 
b ~ normal(0, 2.5)
Intercept ~ normal(0, 2.5)
<lower=0> sd ~ cauchy(0, 2.5)

Group-Level Effects: 
~sub_id (Number of levels: 50) 
       Estimate Est.Error l-95% CI u-95% CI Rhat Bulk_ESS Tail_ESS
sd(x1)     0.45      0.37     0.02     1.32 1.00     1280     1946

Population-Level Effects: 
             Estimate Est.Error l-95% CI u-95% CI Rhat Bulk_ESS Tail_ESS
Intercept[1]     0.02      0.17    -0.31     0.36 1.00     7230     4660
Intercept[2]     0.70      0.18     0.36     1.06 1.00    13469     6394
Intercept[3]     1.28      0.20     0.90     1.69 1.00    10842     5492
Intercept[4]     1.46      0.21     1.05     1.91 1.00    10252     5280
Intercept[5]     2.52      0.44     1.79     3.68 1.00     5492     4294
x1              -0.13      0.26    -0.68     0.35 1.00     3843     2582
x2_c             0.27      0.60    -0.92     1.48 1.00     6769     5130
x1:x2_c         -0.16      0.87    -1.89     1.63 1.00     5598     5040

Family Specific Parameters: 
     Estimate Est.Error l-95% CI u-95% CI Rhat Bulk_ESS Tail_ESS
disc     1.00      0.00     1.00     1.00   NA       NA       NA

Draws were sampled using sample(hmc). For each parameter, Bulk_ESS
and Tail_ESS are effective sample size measures, and Rhat is the potential
scale reduction factor on split chains (at convergence, Rhat = 1).
```

###### Confirm convergence: \(\hat R\)

- \(\hat R\)s of all parameters did not exceed 1.1.

```
bayesplot::mcmc_rhat(
  brms::rhat(fit_open_y6_hC_0807)
  )
```

```
Warning: Dropped 1 NAs from 'new_rhat(rhat)'.
```

###### Confirm convergence: Traceplots

```
plot(
  fit_open_y6_hC_0807, 
  variable = "^(b_x|sd_).*$", 
  regex = T, ask = F
  )
```

```
plot(
  fit_open_y6_hC_0807, 
  variable = "^b_Intercept\\[\\d+\\]", 
  regex = T, ask = F
  )
```

###### Summary

```
summary(
  fit_open_y6_hC_0807,
  priors = TRUE,
  prob = 0.95,  
  robust = TRUE
  )
```

```
Warning: There were 1 divergent transitions after
warmup. Increasing adapt_delta above 0.8 may help. See
http://mc-stan.org/misc/warnings.html#divergent-transitions-after-warmup
```

```
 Family: cumulative 
  Links: mu = probit; disc = identity 
Formula: y6 ~ x1 * x2_c + (0 + x1 | sub_id) 
   Data: dat4fit_open (Number of observations: 116) 
  Draws: 4 chains, each with iter = 4000; warmup = 2000; thin = 1;
         total post-warmup draws = 8000

Priors: 
b ~ normal(0, 2.5)
Intercept ~ normal(0, 2.5)
<lower=0> sd ~ cauchy(0, 2.5)

Group-Level Effects: 
~sub_id (Number of levels: 58) 
       Estimate Est.Error l-95% CI u-95% CI Rhat Bulk_ESS Tail_ESS
sd(x1)     0.30      0.26     0.01     0.92 1.00     1746     3163

Population-Level Effects: 
              Estimate Est.Error l-95% CI u-95% CI Rhat Bulk_ESS Tail_ESS
Intercept[1]     -0.07      0.16    -0.39     0.24 1.00     8923     5274
Intercept[2]      0.20      0.16    -0.12     0.51 1.00    11041     5804
Intercept[3]      0.53      0.16     0.21     0.85 1.00    12508     6172
Intercept[4]      0.69      0.17     0.37     1.02 1.00    12036     6018
Intercept[5]      0.91      0.17     0.58     1.24 1.00    11341     6436
Intercept[6]      1.07      0.17     0.73     1.42 1.00    10606     6286
Intercept[7]      1.16      0.17     0.81     1.51 1.00     9750     6179
Intercept[8]      1.34      0.19     0.98     1.71 1.00     9552     5864
Intercept[9]      1.52      0.19     1.15     1.90 1.00     9281     5489
Intercept[10]     1.88      0.22     1.47     2.31 1.00     8247     6098
x1                0.55      0.21     0.14     0.97 1.00    10079     5591
x2_c              0.09      0.46    -0.81     1.01 1.00     6004     5330
x1:x2_c          -0.09      0.65    -1.36     1.19 1.00     5727     5313

Family Specific Parameters: 
     Estimate Est.Error l-95% CI u-95% CI Rhat Bulk_ESS Tail_ESS
disc     1.00      0.00     1.00     1.00   NA       NA       NA

Draws were sampled using sample(hmc). For each parameter, Bulk_ESS
and Tail_ESS are effective sample size measures, and Rhat is the potential
scale reduction factor on split chains (at convergence, Rhat = 1).
```

```
formula_y7 <-
   bf(
     y7 ~ x1*x2_c + (0 + x1 | sub_id), 
     family = cumulative("probit")
     )
prior_otherItems <- 
  prior(normal(0, 2.5), class = "b") +
  prior(normal(0, 2.5), class = "Intercept") +
  prior(cauchy(0, 2.5), class = "sd")

fit_our_y7_hC_0716 <- 
  brm(
    formula = formula_y7,
    data = dat4fit_our,
    prior = prior_otherItems,
    seed = 1, iter = 4000, warmup = 2000,
    file = "fit_our_y7_0716",
    file_refit = "never",
    refresh = 2000
    )

fit_open_y7_hC_0807 <- 
  update(
    fit_our_y7_hC_0716,
    newdata = dat4fit_open,
    seed = 1,
    file = "fit_open_y7_0807",
    file_refit = "never",
    refresh = 2000
    )
```

- Our data
- Open data

###### Confirm convergence: \(\hat R\)

- \(\hat R\)s of all parameters did not exceed 1.1.

```
bayesplot::mcmc_rhat(
  brms::rhat(fit_our_y7_hC_0716)
  )
```

```
Warning: Dropped 1 NAs from 'new_rhat(rhat)'.
```

###### Confirm convergence: Traceplots

```
plot(
  fit_our_y7_hC_0716, 
  variable = "^(b_x|sd_).*$", 
  regex = T, ask = F
  )
```

```
plot(
  fit_our_y7_hC_0716, 
  variable = "^b_Intercept\\[\\d+\\]", 
  regex = T, ask = F
  )
```

###### Summary

```
summary(
  fit_our_y7_hC_0716,
  priors = TRUE,
  prob = 0.95,  
  robust = TRUE
  )
```

```
Warning: There were 6 divergent transitions after
warmup. Increasing adapt_delta above 0.8 may help. See
http://mc-stan.org/misc/warnings.html#divergent-transitions-after-warmup
```

```
 Family: cumulative 
  Links: mu = probit; disc = identity 
Formula: y7 ~ x1 * x2_c + (0 + x1 | sub_id) 
   Data: dat4fit_our (Number of observations: 100) 
  Draws: 4 chains, each with iter = 4000; warmup = 2000; thin = 1;
         total post-warmup draws = 8000

Priors: 
b ~ normal(0, 2.5)
Intercept ~ normal(0, 2.5)
<lower=0> sd ~ cauchy(0, 2.5)

Group-Level Effects: 
~sub_id (Number of levels: 50) 
       Estimate Est.Error l-95% CI u-95% CI Rhat Bulk_ESS Tail_ESS
sd(x1)     0.45      0.35     0.03     1.27 1.00     1264     2082

Population-Level Effects: 
             Estimate Est.Error l-95% CI u-95% CI Rhat Bulk_ESS Tail_ESS
Intercept[1]    -0.33      0.17    -0.66     0.02 1.00     6246     4646
Intercept[2]     0.20      0.17    -0.12     0.52 1.00     9273     5850
Intercept[3]     0.61      0.18     0.28     0.96 1.00     8914     5829
Intercept[4]     0.90      0.18     0.55     1.27 1.00     7661     5517
Intercept[5]     1.48      0.22     1.06     1.94 1.00     5752     4921
x1               0.04      0.23    -0.43     0.50 1.00     6322     4586
x2_c            -0.02      0.56    -1.12     1.11 1.00     6149     4832
x1:x2_c          0.16      0.82    -1.52     1.80 1.00     5237     5195

Family Specific Parameters: 
     Estimate Est.Error l-95% CI u-95% CI Rhat Bulk_ESS Tail_ESS
disc     1.00      0.00     1.00     1.00   NA       NA       NA

Draws were sampled using sample(hmc). For each parameter, Bulk_ESS
and Tail_ESS are effective sample size measures, and Rhat is the potential
scale reduction factor on split chains (at convergence, Rhat = 1).
```

###### Confirm convergence: \(\hat R\)

- \(\hat R\)s of all parameters did not exceed 1.1.

```
bayesplot::mcmc_rhat(
  brms::rhat(fit_open_y7_hC_0807)
  )
```

```
Warning: Dropped 1 NAs from 'new_rhat(rhat)'.
```

###### Confirm convergence: Traceplots

```
plot(
  fit_open_y7_hC_0807, 
  variable = "^(b_x|sd_).*$", 
  regex = T, ask = F
  )
```

```
plot(
  fit_open_y7_hC_0807, 
  variable = "^b_Intercept\\[\\d+\\]", 
  regex = T, ask = F
  )
```

###### Summary

```
summary(
  fit_open_y7_hC_0807,
  priors = TRUE,
  prob = 0.95,  
  robust = TRUE
  )
```

```
 Family: cumulative 
  Links: mu = probit; disc = identity 
Formula: y7 ~ x1 * x2_c + (0 + x1 | sub_id) 
   Data: dat4fit_open (Number of observations: 116) 
  Draws: 4 chains, each with iter = 4000; warmup = 2000; thin = 1;
         total post-warmup draws = 8000

Priors: 
b ~ normal(0, 2.5)
Intercept ~ normal(0, 2.5)
<lower=0> sd ~ cauchy(0, 2.5)

Group-Level Effects: 
~sub_id (Number of levels: 58) 
       Estimate Est.Error l-95% CI u-95% CI Rhat Bulk_ESS Tail_ESS
sd(x1)     0.45      0.35     0.02     1.16 1.00     1039     2307

Population-Level Effects: 
              Estimate Est.Error l-95% CI u-95% CI Rhat Bulk_ESS Tail_ESS
Intercept[1]     -0.24      0.16    -0.56     0.08 1.00     5767     4796
Intercept[2]      0.15      0.16    -0.15     0.46 1.00     8793     5900
Intercept[3]      0.40      0.16     0.09     0.70 1.00    10289     6135
Intercept[4]      0.53      0.16     0.22     0.85 1.00    10956     6315
Intercept[5]      0.73      0.17     0.41     1.05 1.00     9544     6720
Intercept[6]      0.94      0.17     0.60     1.28 1.00     8140     6389
Intercept[7]      1.05      0.18     0.71     1.41 1.00     7082     5839
Intercept[8]      1.20      0.18     0.85     1.57 1.00     5976     5672
Intercept[9]      1.36      0.19     1.00     1.75 1.00     5362     5411
Intercept[10]     1.89      0.23     1.47     2.36 1.00     4047     4102
x1                0.50      0.21     0.08     0.92 1.00     6971     5902
x2_c              0.23      0.46    -0.66     1.10 1.00     6066     6057
x1:x2_c          -0.76      0.67    -2.06     0.52 1.00     4858     5461

Family Specific Parameters: 
     Estimate Est.Error l-95% CI u-95% CI Rhat Bulk_ESS Tail_ESS
disc     1.00      0.00     1.00     1.00   NA       NA       NA

Draws were sampled using sample(hmc). For each parameter, Bulk_ESS
and Tail_ESS are effective sample size measures, and Rhat is the potential
scale reduction factor on split chains (at convergence, Rhat = 1).
```

# 8 About my enviroment

- We will show the results of `sessioninfo::session_info`, but it incorrectly reports that the `os` is Windows 10.
- The correct one is Windows 11, as shown in the following block:

```
system("wmic os get caption", intern = T)
```

```
[1] "Caption                    \r" "Microsoft Windows 11 Home  \r"
[3] "\r"
```

```
sessioninfo::session_info()
```

```
─ Session info ───────────────────────────────────────────────────────────────
 setting  value
 version  R version 4.2.3 (2023-03-15 ucrt)
 os       Windows 10 x64 (build 22621)
 system   x86_64, mingw32
 ui       RTerm
 language (EN)
 collate  Japanese_Japan.utf8
 ctype    Japanese_Japan.utf8
 tz       Asia/Tokyo
 date     2023-10-04
 pandoc   3.1.1 @ C:/Program Files/RStudio/resources/app/bin/quarto/bin/tools/ (via rmarkdown)

─ Packages ───────────────────────────────────────────────────────────────────
 package        * version  date (UTC) lib source
 abind            1.4-5    2016-07-21 [1] CRAN (R 4.2.0)
 arrayhelpers     1.1-0    2020-02-04 [1] CRAN (R 4.2.3)
 backports        1.4.1    2021-12-13 [1] CRAN (R 4.2.0)
 base64enc        0.1-3    2015-07-28 [1] CRAN (R 4.2.0)
 bayesplot        1.10.0   2022-11-16 [1] CRAN (R 4.2.2)
 bridgesampling   1.1-2    2021-04-16 [1] CRAN (R 4.2.3)
 brms           * 2.19.0   2023-03-14 [1] CRAN (R 4.2.3)
 Brobdingnag      1.2-9    2022-10-19 [1] CRAN (R 4.2.3)
 callr            3.7.3    2022-11-02 [1] CRAN (R 4.2.2)
 checkmate        2.1.0    2022-04-21 [1] CRAN (R 4.2.2)
 cli              3.6.0    2023-01-09 [1] CRAN (R 4.2.2)
 cmdstanr       * 0.5.3    2022-08-01 [1] local
 coda             0.19-4   2020-09-30 [1] CRAN (R 4.2.2)
 codetools        0.2-19   2023-02-01 [2] CRAN (R 4.2.3)
 colorspace       2.0-3    2022-02-21 [1] CRAN (R 4.2.2)
 colourpicker     1.2.0    2022-10-28 [1] CRAN (R 4.2.2)
 crayon           1.5.2    2022-09-29 [1] CRAN (R 4.2.2)
 crosstalk        1.2.0    2021-11-04 [1] CRAN (R 4.2.2)
 digest           0.6.31   2022-12-11 [1] CRAN (R 4.2.2)
 distributional   0.3.1    2022-09-02 [1] CRAN (R 4.2.2)
 dplyr          * 1.1.2    2023-04-20 [1] CRAN (R 4.2.3)
 DT               0.27     2023-01-17 [1] CRAN (R 4.2.2)
 dygraphs         1.1.1.6  2018-07-11 [1] CRAN (R 4.2.2)
 ellipsis         0.3.2    2021-04-29 [1] CRAN (R 4.2.2)
 evaluate         0.20     2023-01-17 [1] CRAN (R 4.2.2)
 fansi            1.0.3    2022-03-24 [1] CRAN (R 4.2.2)
 farver           2.1.1    2022-07-06 [1] CRAN (R 4.2.2)
 fastmap          1.1.0    2021-01-25 [1] CRAN (R 4.2.2)
 forcats        * 1.0.0    2023-01-29 [1] CRAN (R 4.2.3)
 generics         0.1.3    2022-07-05 [1] CRAN (R 4.2.2)
 ggdist           3.2.1    2023-01-18 [1] CRAN (R 4.2.3)
 ggplot2        * 3.4.2    2023-04-03 [1] CRAN (R 4.2.3)
 glue             1.6.2    2022-02-24 [1] CRAN (R 4.2.2)
 gridExtra        2.3      2017-09-09 [1] CRAN (R 4.2.2)
 gtable           0.3.1    2022-09-01 [1] CRAN (R 4.2.2)
 gtools           3.9.4    2022-11-27 [1] CRAN (R 4.2.2)
 HDInterval       0.2.4    2022-11-17 [1] CRAN (R 4.2.3)
 highr            0.10     2022-12-22 [1] CRAN (R 4.2.2)
 hms              1.1.3    2023-03-21 [1] CRAN (R 4.2.3)
 htmltools        0.5.4    2022-12-07 [1] CRAN (R 4.2.2)
 htmlwidgets      1.6.1    2023-01-07 [1] CRAN (R 4.2.2)
 httpuv           1.6.8    2023-01-12 [1] CRAN (R 4.2.2)
 igraph           1.3.5    2022-09-22 [1] CRAN (R 4.2.2)
 inline           0.3.19   2021-05-31 [1] CRAN (R 4.2.2)
 janeaustenr      1.0.0    2022-08-26 [1] CRAN (R 4.2.3)
 jsonlite         1.8.4    2022-12-06 [1] CRAN (R 4.2.2)
 knitr            1.41     2022-11-18 [1] CRAN (R 4.2.2)
 labeling         0.4.2    2020-10-20 [1] CRAN (R 4.2.0)
 later            1.3.0    2021-08-18 [1] CRAN (R 4.2.2)
 lattice          0.20-45  2021-09-22 [2] CRAN (R 4.2.3)
 lifecycle        1.0.3    2022-10-07 [1] CRAN (R 4.2.2)
 loo              2.5.1    2022-03-24 [1] CRAN (R 4.2.2)
 lubridate      * 1.9.2    2023-02-10 [1] CRAN (R 4.2.3)
 magrittr         2.0.3    2022-03-30 [1] CRAN (R 4.2.2)
 markdown         1.4      2022-11-16 [1] CRAN (R 4.2.2)
 Matrix           1.5-3    2022-11-11 [2] CRAN (R 4.2.3)
 matrixStats      0.63.0   2022-11-18 [1] CRAN (R 4.2.2)
 mime             0.12     2021-09-28 [1] CRAN (R 4.2.0)
 miniUI           0.1.1.1  2018-05-18 [1] CRAN (R 4.2.2)
 munsell          0.5.0    2018-06-12 [1] CRAN (R 4.2.2)
 mvtnorm          1.1-3    2021-10-08 [1] CRAN (R 4.2.0)
 nlme             3.1-162  2023-01-31 [2] CRAN (R 4.2.3)
 openxlsx       * 4.2.5.2  2023-02-06 [1] CRAN (R 4.2.3)
 pillar           1.9.0    2023-03-22 [1] CRAN (R 4.2.3)
 pkgbuild         1.4.0    2022-11-27 [1] CRAN (R 4.2.2)
 pkgconfig        2.0.3    2019-09-22 [1] CRAN (R 4.2.2)
 plyr             1.8.8    2022-11-11 [1] CRAN (R 4.2.2)
 posterior        1.3.1    2022-09-06 [1] CRAN (R 4.2.2)
 prettyunits      1.1.1    2020-01-24 [1] CRAN (R 4.2.2)
 processx         3.8.0    2022-10-26 [1] CRAN (R 4.2.2)
 promises         1.2.0.1  2021-02-11 [1] CRAN (R 4.2.2)
 ps               1.7.2    2022-10-26 [1] CRAN (R 4.2.2)
 purrr          * 1.0.1    2023-01-10 [1] CRAN (R 4.2.3)
 R6               2.5.1    2021-08-19 [1] CRAN (R 4.2.2)
 ragg             1.2.5    2023-01-12 [1] CRAN (R 4.2.3)
 Rcpp           * 1.0.9    2022-07-08 [1] CRAN (R 4.2.2)
 RcppParallel     5.1.7    2023-02-27 [1] CRAN (R 4.2.3)
 readr          * 2.1.4    2023-02-10 [1] CRAN (R 4.2.3)
 reshape2         1.4.4    2020-04-09 [1] CRAN (R 4.2.2)
 rlang            1.1.1    2023-04-28 [1] CRAN (R 4.2.3)
 rmarkdown        2.19     2022-12-15 [1] CRAN (R 4.2.2)
 rstan            2.21.7   2022-09-08 [1] CRAN (R 4.2.2)
 rstantools       2.2.0    2022-04-08 [1] CRAN (R 4.2.2)
 rstudioapi       0.14     2022-08-22 [1] CRAN (R 4.2.3)
 scales           1.2.1    2022-08-20 [1] CRAN (R 4.2.2)
 sessioninfo      1.2.2    2021-12-06 [1] CRAN (R 4.2.3)
 shiny            1.7.4    2022-12-15 [1] CRAN (R 4.2.2)
 shinyjs          2.1.0    2021-12-23 [1] CRAN (R 4.2.2)
 shinystan        2.6.0    2022-03-03 [1] CRAN (R 4.2.2)
 shinythemes      1.2.0    2021-01-25 [1] CRAN (R 4.2.2)
 SnowballC        0.7.1    2023-04-25 [1] CRAN (R 4.2.3)
 StanHeaders      2.21.0-7 2020-12-17 [1] CRAN (R 4.2.2)
 stringi          1.7.12   2023-01-11 [1] CRAN (R 4.2.2)
 stringr        * 1.5.0    2022-12-02 [1] CRAN (R 4.2.2)
 svUnit           1.0.6    2021-04-19 [1] CRAN (R 4.2.3)
 systemfonts      1.0.4    2022-02-11 [1] CRAN (R 4.2.3)
 tensorA          0.36.2   2020-11-19 [1] CRAN (R 4.2.0)
 textshaping      0.3.6    2021-10-13 [1] CRAN (R 4.2.3)
 threejs          0.3.3    2020-01-21 [1] CRAN (R 4.2.2)
 tibble         * 3.2.1    2023-03-20 [1] CRAN (R 4.2.3)
 tidybayes      * 3.0.4    2023-03-14 [1] CRAN (R 4.2.3)
 tidyr          * 1.3.0    2023-01-24 [1] CRAN (R 4.2.3)
 tidyselect       1.2.0    2022-10-10 [1] CRAN (R 4.2.2)
 tidytext       * 0.4.1    2023-01-07 [1] CRAN (R 4.2.3)
 tidyverse      * 2.0.0    2023-02-22 [1] CRAN (R 4.2.3)
 timechange       0.2.0    2023-01-11 [1] CRAN (R 4.2.3)
 tokenizers       0.3.0    2022-12-22 [1] CRAN (R 4.2.3)
 tzdb             0.3.0    2022-03-28 [1] CRAN (R 4.2.3)
 utf8             1.2.2    2021-07-24 [1] CRAN (R 4.2.2)
 vctrs            0.6.3    2023-06-14 [1] CRAN (R 4.2.3)
 withr            2.5.0    2022-03-03 [1] CRAN (R 4.2.2)
 xfun             0.36     2022-12-21 [1] CRAN (R 4.2.2)
 xtable           1.8-4    2019-04-21 [1] CRAN (R 4.2.2)
 xts              0.12.2   2022-10-16 [1] CRAN (R 4.2.2)
 yaml             2.3.6    2022-10-18 [1] CRAN (R 4.2.2)
 zip              2.3.0    2023-04-17 [1] CRAN (R 4.2.3)
 zoo              1.8-11   2022-09-17 [1] CRAN (R 4.2.2)

 [1] C:/Users/toyok/AppData/Local/R/win-library/4.2
 [2] C:/Program Files/R/R-4.2.3/library

──────────────────────────────────────────────────────────────────────────────
```
